# Microbial-geochemical Interactions in Underground Reservoirs: Implications for Hydrogen Storage

**DOI:** 10.1007/s00248-026-02800-8

**Published:** 2026-06-05

**Authors:** Laura Fablet, Arnaud Belcour, Sylvain Stephant, Caroline Michel, Delphine Ropers, Katerina Cerna, Petra Bombach, Jakub Riha, Joachim Tremosa, Biwen Annie An-Stepec, Kristyna Fadrhonc, Nicole Dopffel, Setareh Rad

**Affiliations:** 1 BRGM, 3 Avenue Claude Guillemin, Orléans Cedex 2, 45060 France; 2 Univ. Grenoble Alpes, Inria, Grenoble, 38000 France; 3 Univ. Grenoble Alpes, CNRS, LIPhy, Grenoble, France; 4 NORCE Research AS, Nygardsgaten 112, Bergen, 5008 Norway; 5 Technical University of Liberec, Institute for Nanomaterials, Advanced Technologies and Innovation, Bendlova 7, Liberec, Czechia; 6 Isodetect GmbH, Deutscher Platz 5b, Leipzig, 04103 Germany; 7 Geostock, 2 rue des Martinets, Rueil-Malmaison, 92500 France

**Keywords:** Hydrogen underground storage, Subsurface, Anaerobic microbiology, Hydrogenotrophic microorganisms

## Abstract

Understanding microbial life in deep geological environments is essential for assessing natural biogeochemical processes and their implications for subsurface activities such as underground hydrogen storage. This study investigates the water chemistry, microbial community composition, and metabolic potential of two contrasting geological settings: porous reservoirs and salt caverns. This study is among the first to provide a large-scale comparison of microbial and geochemical processes across reservoirs. Our results show that porous reservoirs, particularly those with carbonate facies, are characterized by carbonate-rich fluids and host anaerobic microbial communities dominated by methanogenic archaea, fermentative bacteria, and acetogens. In contrast, the studied salt caverns contain salt-saturated brines and are dominated by halophilic and chemoorganotrophic microorganisms adapted to oligotrophic, high-salinity conditions. Some reservoirs showed atypical microbial profiles influenced by anthropogenic activities or hydrogeological connections, which alter local geochemistry and microbial structures. This study highlights the influence of salinity, carbon availability, temperature, and human impact on microbial community structure and metabolic functional potential. The presence of hydrogenotrophic microorganisms raises concerns about their potential to consume injected hydrogen and alter gas quality. These findings underscore the need to move beyond detection toward activity-based assessments of microbial hydrogen consumption, in order to anticipate biogeochemical dynamics and develop effective mitigation strategies. A better understanding of the interplay between reservoir chemistry, geology, and microbiology is crucial for ensuring safe and efficient underground hydrogen storage.

**Supplementary Information:**

The online version contains supplementary material available at 10.1007/s00248-026-02800-8.

## Introduction

The transition to renewable energy sources, essential to meet the challenge of climate change, requires the development of efficient energy storage solutions to overcome their intermittency [1]. Among the available options, hydrogen (H_2_) is particularly attractive for long-term storage. Surplus renewable energy can be converted into H_2_ by electrolysis [2, 3], stored for long periods [4], and then withdrawn for use in chemical industry, mobility or energy systems when demand exceeds supply. Underground Hydrogen Storage (UHS) represents a promising storage option, allowing the temporary storage of large quantities of H_2_ in geological reservoirs. Depending on the type of geological formation, several underground storage systems are distinguished: (1) deep aquifers; (2) (depleted-) hydrocarbon reservoirs, both are porous reservoirs, and (3) salt caverns [5]. Deep aquifers are deep porous formations saturated with brine and generally covered by low-permeability cap rocks that ensure gas confinement, while (depleted-) hydrocarbon reservoirs are former oil or gas fields sealed by impermeable cap rocks. Salt caverns, in contrast, are artificial cavities created within thick salt deposits by solution mining [6]. Once injected, H_2_ comes into contact with the geological environment, where complex water-rock-gas interactions may occur. These processes control the evolution of fluid chemistry (e.g. concentrations of Na^+^, Cl^−^, SO_4_^2−^, Ca^2+^, etc.), porosity changes, mineral phase stability (e.g. halite, calcite, silicates, anhydrite, etc.), and reaction with organic matter, resulting in a diversity of geochemical signatures [7–9]. Several pilot projects have already investigated the feasibility of underground H_2_ storage, for example the Diadema project in depleted gas field [10, 11] or the Underground Sun storage project in a former gas reservoir in the Austrian Molasse Basin [12, 13]. These pilot projects suggest that H_2_ storage is possible on a small scale, but pure H_2_ storage has not yet been demonstrated on a commercial scale. Other pilot projects on H_2_ storage in salt caverns have concluded that this type of reservoir appears to be a more favorable option [14, 15].

Besides abiotic reactions, geological reservoirs also host diverse and metabolically active microbial communities that can impact the fate of stored H_2_ [16–18]. These microorganisms play key roles in the biogeochemical cycles, including carbon, sulfur, nitrogen, iron and trace elements cycles [19], thereby influencing mineral stability and fluid composition [20, 21]. Subsurface microbial communities are typically anaerobic and can tolerate extreme conditions of high salinity (up and beyond 250–300 g/L), temperature (up to 121 °C), and pressure (up and beyond 100–150 MPa), reflecting the specific physicochemical conditions of the deep surface [22–24]. In the past decade, increasing attention has been given to microbiological processes in the context of H_2_ storage [25–27]. Despite their importance, our knowledge of these microbial communities remains limited due to the challenges associated with accessing and sampling these environments [28, 29]. Where studies exist, they frequently reveal a high proportion of unknown or unclassified genera, highlighting the unique and poorly understood biodiversity of subsurface habitats [30–32].

The microbial community composition has been shown to correlate with the profiles of available electron donors and acceptors [33]. Specifically, sulfate-reducing microbes can reduce sulfate (SO_4_^2−^) or other sulfides (HS^−^, S_2_^−^) to the toxic gas H_2_S [34], leading to mineral precipitation (e.g. FeS), and souring of stored H_2_ [18]. Further, hydrogenotrophic methanogenic archaea can consume injected H_2_ and carbon dioxide to produce CH_4_, potentially reducing the efficiency of H_2_ storage [35, 36]. Microbial growth and activity can also induce corrosion processes, biofilm formation and mineral precipitation, potentially affecting injectivity, reservoir porosity and the long-term integrity of the infrastructure [18]. For example, Dohrmann and Krüger (2023) examined the potential for H_2_ consumption by gas reservoir microorganisms at high pressure conditions, and showed that H_2_ is a major energy substrate, potentially leading to H_2_ losses and sulphide production [37]. Mura et al. (2025) found that microbial H_2_ consumption in deep aquifers resulted in alkalinisation. The pH increase led to microbial growth and respiration inhibition, suggesting that after an initial decrease in H_2_ concentration, gas storage could stabilize in deep aquifers [38]. It is therefore essential to understand the coupled geochemical and microbial dynamics in underground storage environments to predict the fate of H_2_, assess the risks associated with storage and ensure the long-term performance of underground H_2_ storage systems.

In this work, the composition and diversity of microbial communities associated with various types of gas storage facilities (porous reservoirs and salt caverns) were determined, exploring in particular their H_2_ consumption potential. It is known that the fluids from porous reservoirs and salt caverns have distinct chemical compositions, which may influence the microbial communities they harbor differently. For this, 19 European underground reservoirs, theoretically suitable for H_2_ storage, were characterized from a chemical and microbial perspective. By combining chemical profiling with microbial community analyses, the interactions between water chemistry and microbial community structures were analyzed to better understand their mutual influences and potential implications for the stability and reactivity of these reservoirs.

## Materials & Methods

### Reservoir Characteristics

**Fig. 1:**
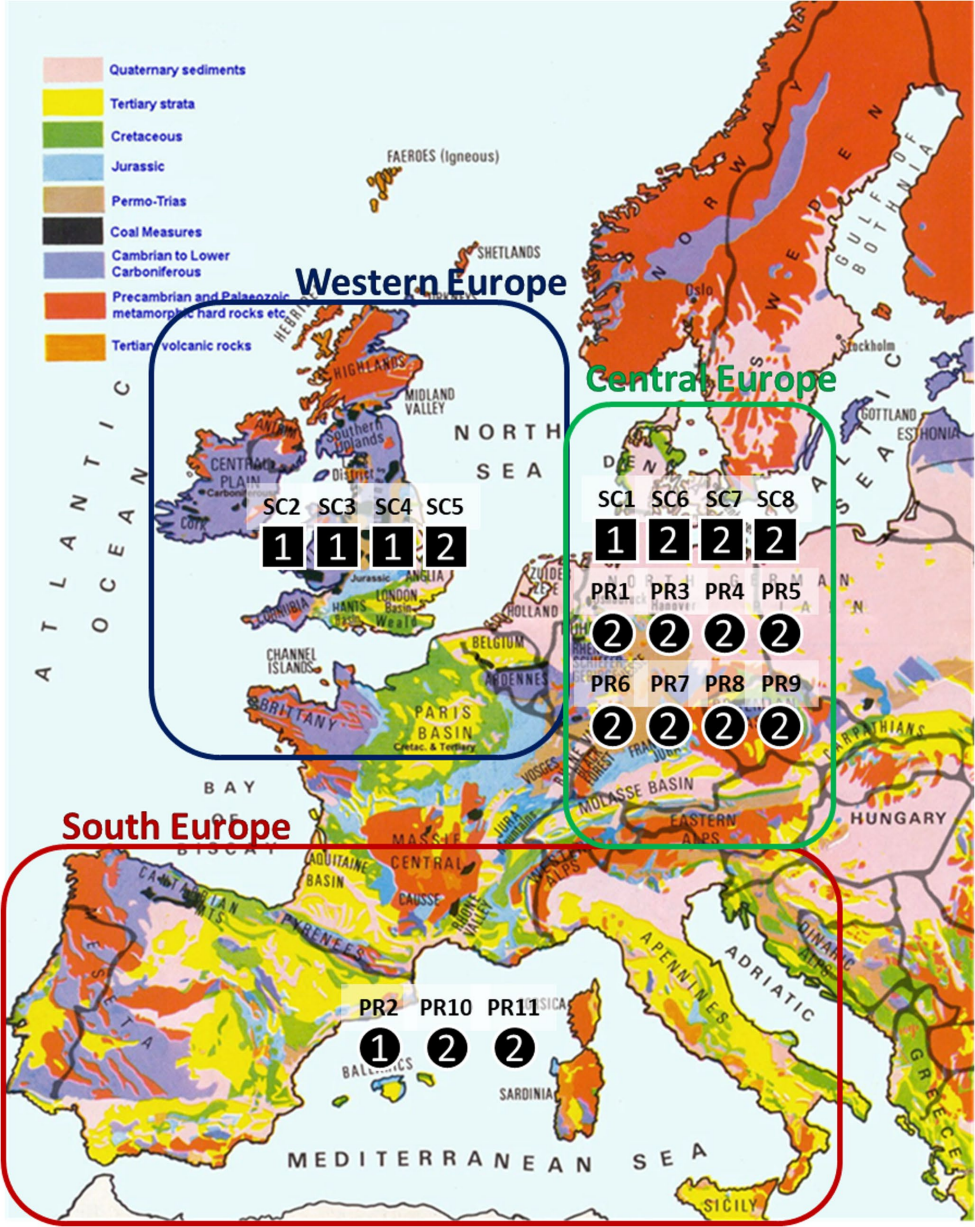
Location of sampled reservoirs on the geological map of Europe (modified from West, 2012). The sampling was realised in three areas in Europe: western Europe in blue (SC2, S3, SC4 and SC5), central Europe in green (SC1, SC6, SC7, SC8, PR1, PR3, PR4, PR5, PR6, PR7, PR8 and PR9), and south Europe in red (PR2, PR10 and PR11) The positions of samples are approximate and should not be interpreted as exact. The shape of symbols indicates the type of reservoir: squares for salt caverns (SC) and circles for porous reservoirs (PR). The number of circles corresponds to the number of sampled reservoirs and the number in the circles indicates the number of samples taken for the sequencing data

The reservoirs studied are located in different European countries in south Europe, central Europe and western Europe. A total of nineteen different reservoir samples were collected in salt caverns and depleted-hydrocarbon reservoirs, the latter being referred to as porous reservoirs in this article (Fig. 1). Eleven porous reservoirs were sampled: eight in central Europe (PR1, PR3 – PR9) and three in south Europe (PR2, PR10 and PR11). Four salt caverns were sampled in western Europe (SC2 – SC5) and four in central Europe (SC1, SC6 - SC8). The characteristics and specific information about the reservoirs are indicated in Table 1. Six of these reservoirs are knowingly affected by recent anthropogenic activities: (i) PR5 was contaminated by accidental lubricant injection, (ii) SC3 brine contains dissolved ethylene gas, and (iii) an anticorrosive agent (sodium hydroxide) was injected into SC6, SC7 and SC8. The sampled reservoirs have varying depths, ranging from 300 to 2300 m. The depths of PR8 and PR9 are unknown. In our samples, porous reservoirs present a large range of salinity, from 0.08 to 176 g/L in porous reservoirs, while salt caverns have salinities between 303 and 404 g/L in the salt caverns. For simplicity, the general term “reservoir fluids” is used for all water samples in the rest of the text. Samples also exhibited a wide temperature range, from 15 to 48 °C for salt caverns and from 13 to 150 °C for porous reservoirs.

**Table 1:** Characteristics of samples collected and specific information about reservoirs: anthropic impacts, approximate reservoir depth (m), salinity (g/L) of reservoir fluids, ionic balance error (%) and temperature of reservoirs. SC = Salt cavern and PR = Porous reservoir

Sample	Europe area	Recent anthropic impacts	Reservoir depth (m)	Salinity (g/L)	Ionic balance error (%)	Assumed temperature
PR1	Central Europe	Not known	623	20	-10.36	13 °C
PR2	South Europe	Not known	2300	13	4.68	90 °C
PR3	Central Europe	Not known	1420	11	3.36	49 °C
PR4	Central Europe	Not known	1495	17	-3.18	47 °C
PR5	Central Europe	Lubricant injection	1500	80	3.43	44 °C
PR6	Central Europe	Not known	625	2.4	-9.7	41 °C
PR7	Central Europe	Driven by a saline aquifer from beneath	Unknown	176	-0.03	150 °C
PR8	Central Europe	Not known	Unknown	0.1	-4.62	150 °C
PR9	Central Europe	Not known	Unknown	11	1.42	39 °C
PR10	South Europe	Not known	956	87	-4.99	61 °C
PR11	South Europe	Not known	965	86	-4.59	61 °C
SC1	Central Europe	Not known	1310	331	-0.34	48 °C
SC2	Western Europe	Not known	409	307	-1.02	18 °C
SC3	Western Europe	Presence of ethylene	471	303	-1.28	18 °C
SC4	Western Europe	Not known	317	319	-4.05	15 °C
SC5	Western Europe	Not known	1199	404	-4.59	21 °C
SC6	Central Europe	Injection of anticorrosive agent	894	308	-8.13	30 °C
SC7	Central Europe		1225	307	-4.25	30 °C
SC8	Central Europe		956	303	-3.16	30 °C

### Reservoir Fluid Sampling

The sampling method (wellhead, gas separator or downhole) applied for each reservoir is indicated in Table 2. A wellhead sample is a water sample taken from a sampling tap located at the wellhead. In this case, reservoir fluid is pumped and sampled as it rises to the surface. A water sample taken after the gas separator corresponds to cases in which gas, which was present at high pressure in the reservoir, must be removed from the water before it can be safely sampled. After the gas separator, the water sample is outgassed and flows through the surface installation after being pumped from the well. A downhole sample is taken with a sterilized sampler lifted down the well to the target depth with a slickline or wireline control. With a downhole sample, water is sampled directly in the reservoir, at the depth of the well perforation, or in the salt cavern. All sampling equipment was disinfected with 70% ethanol and/or sterilized at 121 °C beforehand. Prior to surface sampling, the water was drained for 5 to 10 min to eliminate stagnant water. Water was collected in 1 L bottles previously flushed with sterile N_2_ gas. A permanent N_2_ flush was maintained throughout sampling. The bottles were filled with 80% reservoir fluid, then flushed with N_2_ for 2–3 min to eliminate any oxygen present. Bottles were sealed with sterile thick caps to prevent oxygen contamination and immediately sent to the lab. During shipping, samples were stored at room temperature in polystyrene boxes to protect them from light. The collected water was then used for chemical analysis. For the determination of microbial communities based on DNA, at least 100 mL of water (depending on sampling conditions such as flow rate and operating time) was filtered through Millipore Sterivex 0.22 µM filters directly at site to avoid changing community patterns during transport, from pre-filled bottles. The Sterivex filters were fixed with 70% isopropanol, closed, and shipped at ambient temperatures to the four laboratories of the project, for DNA extraction, before to be send to one lab for the sequencing. A detailed reservoir fluid sampling procedure and its standardization are described in Cerna et al. (2025) [39].

**Table 2:** Sampling method for each reservoir: wellhead, gas separator above ground or downhole. Wellhead corresponds to a water sample taken directly from the wellhead. Gas separator corresponds to a water withdrawal after the well fluid reaches the surface and is separated from the gas phase. Downhole corresponds to a direct water extraction from the geological formation at the bottom of the borehole

Sample	Sampling method	Remarks
PR1	Downhole	
PR2	Gas separator	O_2_ exposure during sampling
PR3	Downhole	
PR4	Downhole	
PR5	Downhole	
PR6	Downhole	
PR7	Gas separator	
PR8	Gas separator	
PR9	Wellhead	
PR10	Wellhead	
PR11	Wellhead	
SC1	Downhole	
SC2	Downhole	
SC3	Downhole	
SC4	Downhole	
SC5	Downhole	
SC6	Wellhead	High volume of brine flowed before sampling *
SC7	Wellhead	High volume of brine flowed before sampling *
SC8	Wellhead	High volume of brine flowed before sampling *

* corresponding to 3 times the volume of the well

### Physico-chemical Analysis

Depending on the laboratory, different methodologies were used, as demonstrated in the study by Cerna et al. (2025) [39]. Anions were quantified by ion or liquid chromatography (Cl^−^, SO_4_^2−^, NO_3_^−^, NO_2_^−^) and spectrophotometry (PO_4_^3−^). Cation analyses were performed by ICP-OES/AES (Na^+^, Ca^2+^, Mg^2+^, K^+^, Fe_total_, Mn^2+^) and spectrophotometry (NO_2_, NH_4_). Quantification limits vary from 0.1 to 20 mg/L for cations and from 0.01 to 50 mg/L for anions, depending on the element measured. Carbonates (HCO_3_ and CO_3_) were analyzed by potentiometry (LQ = 10 mg/L). Salinity, conductivity and pH measurements were analyzed using HORIBA LAQUATWIN^®^ probes in the lab. To verify the consistency of the analytical results, the ionic balance was calculated with PhreeqC, using the Thermodemm database [40] for waters with salinity lower than 30 g/L and Pitzer database [41] for waters with salinity higher than 30 g/L (Table 1). Samples had a relative ion balance error of up to 10%. Considering the high dilutions applied and low concentrations measured in some cases, the sensitivity to analytical errors increased. Therefore, the error margin of up to 10% is acceptable, particularly given the high salinities of the water.

### DNA Extraction and Sequencing

Depending on the site, one or two samples were taken for sequencing (Fig. 1). For DNA extraction from Sterivex filters [42, 43], DNeasy^®^ Blood & Tissue Kit (Qiagen) was used. The first step of cell lysis took place directly in the filter capsule by adding 1800 µL of ATL buffer and 200 µL of proteinase K. Lysis was carried out at 56 °C for around 16 h. At the end of this step, the lysed filter contents were transferred to a clean tube with 900 µL AL buffer and 900 µL absolute ethanol. The mixture was then transferred to a DNAeasy mini-centrifuge and DNA was extracted based on the manufacturers protocol. Samples were then stored at 6 °C or -20 °C. Extraction kit controls (i.e. clean Sterivex that were not submitted to sample filtration) were systematically included to monitor potential contamination during the extraction process. The samples corresponding to these controls were rated from C1 to C14.

To compare microbial composition between reservoirs, 16 S amplicon sequencing (NGS) was performed. Libraries were prepared by two consecutive PCR reactions: the first with standard primers and the second with similar, but barcoded fusion primers. Thermocycling conditions were identical for both PCR setups, except for the number of cycles: an initial denaturation at 95 °C for 3 min; 10 cycles (first PCR) or 35 cycles (second PCR) of denaturation at 98 °C for 20 s, annealing at 50 °C for 15 s, and elongation at 72 °C for 45 s; followed by a final extension at 72 °C for 1 min. Amplification targeted the hypervariable V4 region of the 16 S rRNA gene using EliZyme HS HIFI MIX polymerase (Elizabeth Pharmacon, Czech Republic) and universal primers 515 F (GTGYCAGCMGCCGCGGTAA) and 806R (GGACTACNVGRGTWTCTAAT). Amplified products were purified (Ampure XP), quantified (Qubit 2.0), and mixed at equimolar concentrations before sequencing on Ion Torrent Genexus System. Raw data were processed using QIIME 2 (2021.8) [44]: demultiplexing, quality filtering (q2-demux), denoising (DADA2) [45], taxonomic assignment via classify-sklearn on the Silva 138 database [46], removing mitochondria and chloroplasts. Classification accuracy was checked on an artificial MOCK community, and results were analyzed using Phyloseq (R) [47]. The sequencing data have been deposited in the NCBI Sequence Read Archive under BioProject accession number [PRJNA1328997] (https:/www.ncbi.nlm.nih.gov/bioproject/PRJNA1328997).

### Prediction of Metabolic Functions in Microbial Communities

The taxonomic compositions and abundances were processed using the Tabigecy workflow version 0.1.1 [48]. Tabigecy relies on EsMeCaTa version 0.6.5 [49] and the EsMeCaTa precomputed database version 1.0.0. EsMeCaTa searches for phylogenically close proteomes associated with the lowest taxonomic affiliation (e.g. genus) provided as input. If no proteomes are available, EsMeCaTa examines higher taxonomic ranks, such as family or class. These proteomes are then searched for metabolic functions in biogeochemical cycles (carbon, sulphur and nitrogen cycles) with bigecyhmm version 0.1.7 [48].

Heatmaps showing the relative abundance of organisms associated with metabolic functions were generated using R version 4.1.2 and ggplot2 version 3.5. Taxa were classified as hydrogen-consuming methanogens if Tabigecy identified both a methanogenesis marker gene (*mcrA*, *mcrB*, or *mcrC*) and methanogenesis-associated hydrogenases (Fe, Ni-Fe 3c, or Ni-Fe 4hi). A heatmap of these results was generated using seaborn (v0.13.2).

### Statistical Analyses

All statistical analyses were performed using R 4.4.2.

To assess the reproducibility of biological replicates, Bray-Curtis dissimilarities were computed between all pairs of replicates, and the distribution of intra-sample distances (within replicates) was compared to inter-sample distances (between different samples) using a Wilcoxon rank-sum test. Non-metric multidimensional scaling (NMDS) ordination was then performed on the resulting Bray-Curtis distance matrix to visualize the relationship among replicates.

Sampled sites were clustered according to their chemical composition using k-means clustering. The optimal number of clusters was determined by the Elbow method and silhouette method [50], leading to the identification of four chemical clusters. To further explore chemical patterns among samples, a Principal Component Analysis (PCA) was performed to highlight the main gradients of chemical variations between samples [51].

Microbial community structures across sites were further analyzed using NMDS on Bray-Curtis dissimilarities, followed by k-means clustering. Differences in microbial community composition between clusters were assessed using ANOSIM. Alpha diversity (species richness, Shannon, Simpson) was calculated for each cluster, and differences were evaluated using Kruskal-Wallis tests, followed by post-hoc comparisons. To characterize the structure of microbial communities within clusters, dominant and indicator genera were identified. Dominant genera were defined as those with the highest relative abundance within each cluster, identified separately for Bacteria and Archaea, based on the ten most abundant genera per cluster. Indicator genera are specific to a given environment and were identified using indicator value analysis based on their preferential association with specific clusters, considering both their frequency and specificity within clusters, using “indicspecies” package. They were identified using sample-level data, ensuring that both abundance and occurrence across samples contributed to their selection.

Relationships between microbial communities and environmental variables were further explored using Redundancy analysis (RDA) and Spearman correlations. The RDA, such as PCA, is based on chemical parameters, and enables the exploration of microbial community structure and the distribution of genera along these chemical gradients. In order to visualize the correspondences between chemical and microbiological characteristics, a Sankey diagram was drawn [52].

## Results

### Hydrochemical Characteristics of Formation Reservoir Fluid

**Fig. 2:**
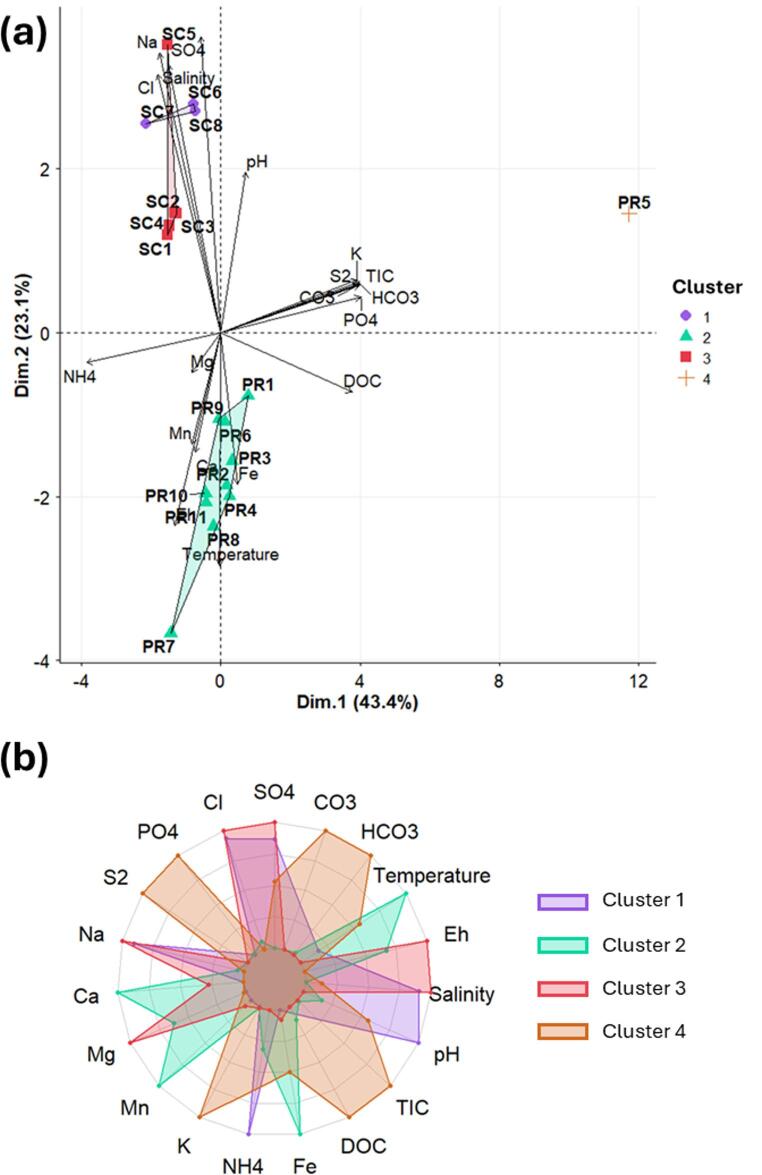
Chemical characterization of reservoir fluid samples. (**a**) Principal Component Analyses (PCA) biplot of reservoir fluid samples based on their chemical composition and regrouped in four clusters. The principal components 1 and 2, (Dim.1 and Dim.2), explain 43.4% and 23.1% of the total variance, respectively. Arrows indicate the direction and strength of each chemical variable contribution to the principal components. Cluster 1 is represented by purple circles, Cluster 2 by green triangles, Cluster 3 by red squares and Cluster 4 by orange cross. (**b**) Radar chart showing the average chemical composition of samples in each cluster

Sample chemical compositions were analysed and subsequently evaluated using a PCA (Fig. 2a). The k-means results groups the sites into four clusters, taking into account pH, Eh, salinity, and temperature, in addition to chemical ones (SO_4_^2−^, Cl^−^, PO_4_^3−^, NO_3_^−^, NO_2_^−^, S_2_^−^_,_ Na^+^, Ca^2+^, Mg^2+^, Mn^2+^, K^+^, NH_4_^+^, Fe_total_, HCO_3_^−^, CO_3_^2−^, TIC, DOC), summarized in Table S1. The first two principal components (Dim.1 and Dim.2) explain 66.5% of the total variance: Dim.1 = 43.4% and Dim.2 = 23.1%. The overall strength of correlations between samples (PCA axis scores and their physicochemical parameters) is summarized in Table S2. PO_4_^3−^, S_2_^−^, NH_4_^+^, HCO_3_^−^, CO_3_^2−^ and TIC showed a significant positive correlation with PC1, while PC2 had a positive correlation with SO_4_^2−^, Cl^−^, Na^+,^ and salinity, as well as temperature and Fe_total_, but to a lesser extent. Specifically, PC1 isolated the PR5 site, while PC2 separated Clusters 1 (SC6, SC7 and SC8) and 3 (SC1, SC2, SC3, SC4 and SC5) on one side, and Cluster 2 (PR1 to PR7 and PR9 to PR11) on the other side. Cluster 1 is clearly separated from Cluster 3 due to its higher pH values, which are captured along dimension 3 in the PCA (Fig. S1). The radar chart (Fig. 2b) shows the four clusters characteristics: (i) Cluster 1 is characterized by high concentrations of Na^+^, Cl^−^, SO_4_^2−^ and NH_4_ with a very high pH, (ii) Cluster 2 is composed of high concentrations of Fe_total_, Mn^2+^, Ca^2+^ with higher temperatures, (iii) Cluster 3 is also defined by high concentrations of Na^+^, Cl^−^ and SO_4_^2−^, but also Mg with high salinity and redox potential (Eh), and (iv) Cluster 4, made up of a single site, has high concentrations of carbon (TIC, DOC, CO_3_^2−^, HCO_3_^−^), PO_4_^3−^, S^2−^ and K^+^. The average concentrations of each element for the four clusters are shown in Fig. S2. A finer scale distribution of the reservoirs is shown in Fig. S3, separating the PR and SC. At the PR level, these results highlight the high salinity of PR7, the high Mg concentrations of PR10 and PR11, and the high temperatures of PR2, PR7 and PR8. Regarding the SC, SC1 has the highest temperature with high carbon concentrations, while SC2 and SC3 have low carbon concentrations, and SC4 has high cation concentrations.

### Structure and Composition of Reservoir Fluid Microbial Communities

#### Microbial Community Structuration

**Fig. 3:**
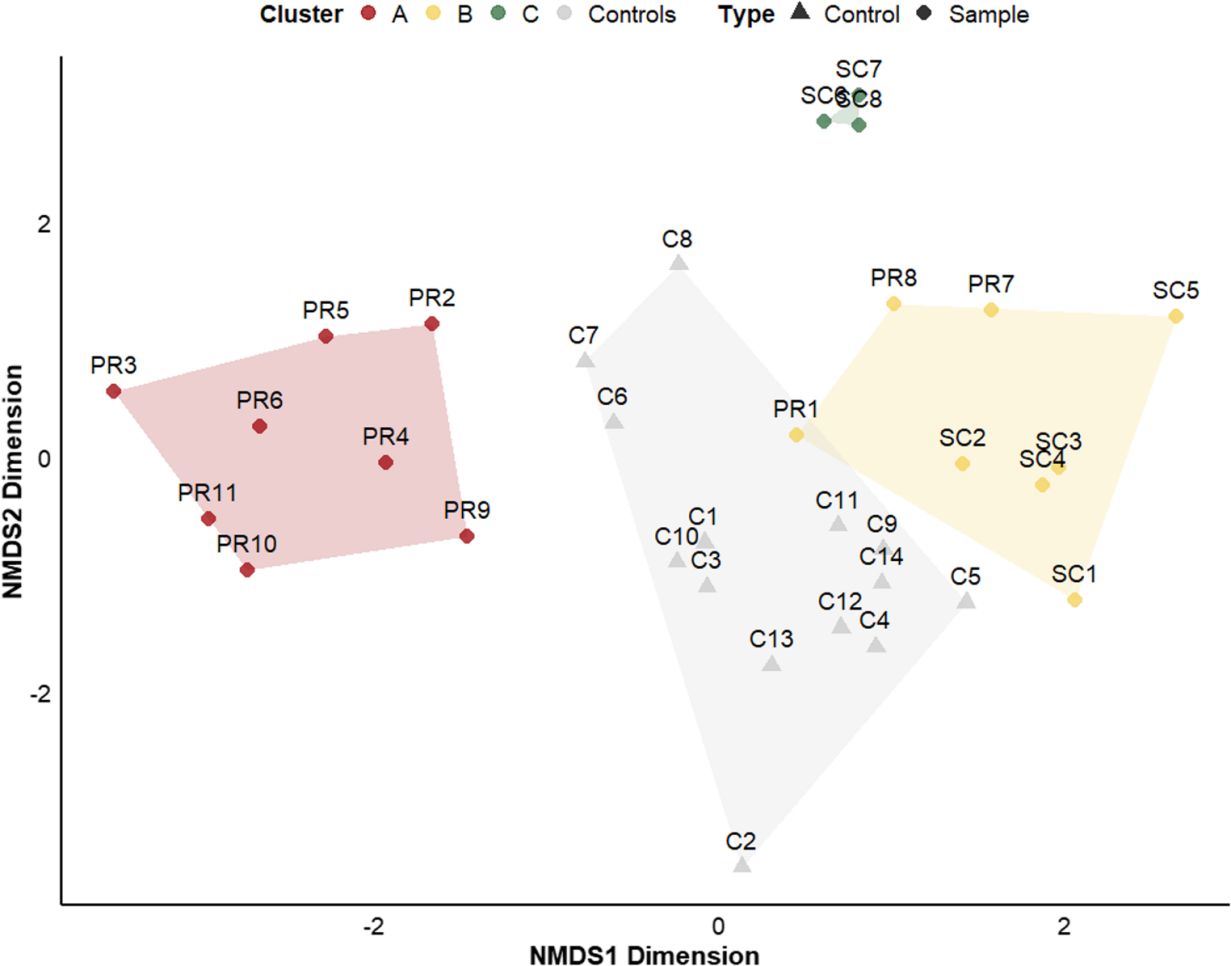
Microbial characterization of reservoir fluid samples with a non-metric multidimensional scaling (NMDS) plot showing microbial community for each site, with the associated clustering: Cluster A (red), Cluster B (yellow), Cluster C (green) and Controls (grey), determined by k-means test

**Fig. 4:**
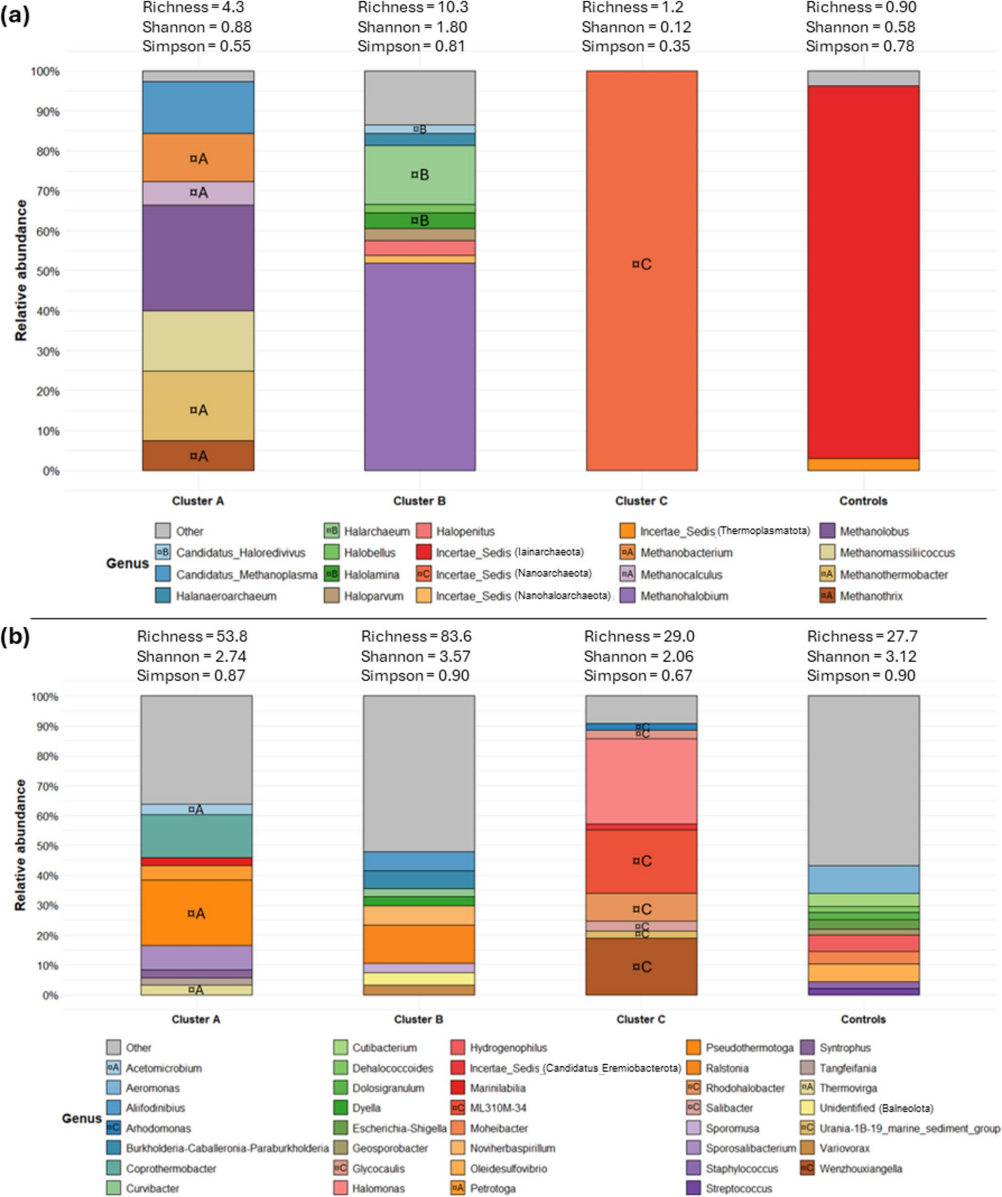
Most abundant microorganisms (> 2%) for each cluster (Cluster A, B and C, and controls) with (**a**) archaeal and (**b**) bacterial genera. The dominant genera which are also indicator genera are marked with a “¤” for each cluster: “¤A” for Cluster A, “¤B” for Cluster B, and “¤C” for Cluster B. No indicator genera are found for controls. Indicator taxa were identified using indicator species analysis. Only taxa with p-values < 0.05 were considered significant for archaea and < 0.01 for bacteria. For each cluster, the average richness, average Shannon index, and average Simpson index are indicated for Archaea and Bacteria

Replicates per sample were averaged after calculating Bray-Curtis distances between replicates (Table S3, Fig. S4), in order to verify the similarity of microbial composition between replicates of the same sample. Intra-sample distances (between replicates of the same samples) are significantly smaller than inter-sample distances (between replicates of different samples), as determined by a Wilcoxon test (*p* < 0.001). The proximity of replicates for the same sample in ordered space strongly suggests the stability of their microbial composition, but also confirms that all steps of the protocol are repeatable from sampling to DNA sequencing. This confirms the consistency of the replicates and justifies their grouping by averaging in subsequent analysis. Sequencing revealed the presence of numerous microbial genera in the reservoir fluid samples (*n* = 1131), and 6.4% of which correspond to unknown genera (“Undetermined” and “*Incertae* Sedis”), referred to in this case by their phylum. A representation of the relative abundance of microbial classes present in each sampled underground water is shown in Fig. S5. The k-means used before NMDS analysis shows a structuring of the microbial communities with three main clusters and a distinct cluster for the kit controls. The results of NMDS analysis are presented in Fig. 3. Cluster A groups PR2, PR3, PR4, PR5, PR6, PR9, PR10 and PR11. Cluster B is made up of samples SC1, PR1, SC2, SC3, SC4, SC5, PR7 and PR8, and represents a second distinct microbiological profile. Cluster C is a small group with three sites: SC6, SC7, and SC8. Finally, the “controls” cluster is relatively isolated from the other clusters, suggesting a different microbial signature, although there is a slight overlap with PR1, which may reflect a sampling or DNA extraction issue in this sample. The resulting weak genetic signal likely captured predominantly background contamination originating from laboratory or extraction kits rather than the true reservoir microbiome. To statistically test the separation observed in the NMDS, an ANOSIM test was performed on the same matrix data. A first analysis comparing environmental samples and controls revealed a significant compositional difference (*R* = 0.1727, *p* = 0.009), indicating that environmental samples carried a biological signal distinct from background contamination. When controls were included in the group comparison, the ANOSIM remained statistically significant (*R* = 0.1269, *p* = 0.032), although the low R value indicates weak group separation. This likely results from the high compositional heterogeneity of control samples, which reflects stochastic background contamination from laboratory reagents rather than a consistent biological signal, thereby increasing within-group dispersion and reducing the overall strength of separation. When controls were excluded and only environmental samples were considered, group separation was strong and highly significant (*R* = 0.875, *p* = 0.001), supporting the ecological relevance of the three identified clusters. The inclusion of controls therefore obscures community differentiation among samples due to their non-ecological and variable composition. The mean specific richness of Cluster B was the highest for both Bacteria and Archaea, but was only significantly different from Cluster C for Archaea, and from the Cluster Controls for Bacteria (Fig. S6a). Concerning diversity index, Clusters A and B have a Shannon index significantly different from the Controls for Archaea, whereas Cluster C has a Shannon index and a Simpson index significantly different from the Controls for Bacteria (Fig. S6b, c). The average values of richness, Simpson index and Shannon index per cluster can be found in Fig. 4. To assess the potential impact of laboratory contaminants on microbial community composition, the decontamination prevalence method was applied using kit controls (Cluster Controls) as negative controls. This analysis identified 11 genera as potential contaminants, with only *Escherichia-Shigella* present in 8 samples among the identified groups (Table S4).

Sampling depth was also tested, but was not significant (*p-value* = 0.123). In addition, NMDS analyses were further performed for each reservoir type separately to examine the distribution of microbial communities within each group. The SC exhibited very similar communities, although three groups could be distinguished: SC6-8, SC2-5, and SC1 (Fig. S7a). The porous reservoirs presented three subgroups with PR1, PR7, PR8 in a first group, PR2, PR3, PR4, PR5 in a second group, and PR6, PR9, PR10, PR11 in a third group, which agrees with the general grouping profiles, notably with the microbial distinction of PR1, PR7 and PR8 from the other porous reservoirs (Fig. S7b).

#### Identification of Microbial Genera Characteristics of Each Cluster

To better understand the structure and ecological functioning of subsurface microbial communities, the abundance of the genera was averaged by cluster. The most abundant archaeal genera, represented with an abundance superior to 2% for each microbial cluster are presented in Fig. 4a. PR samples of Cluster A harbored predominantly *Methanolobus* (26.6%), *Methanothermobacter* (17.3%), *Methanomassiliicocus* (15.1%), *Candidatus Methanosplasma* (13.0%), *Methanobacterium* (12.0%), *Methanothrix* (7.5%) and *Methanocalculus* (5.9%). *Methanobacterium*, *Methanocalculus*, *Methanothermobacter* and *Methanothrix* were also identified as indicator genera. The other archaean genera, with relative abundance below 2%, represented 2.6% of the total abundance of archaea. Concerning Cluster B, more archaeal genera presented an abundance superior to 2%, with *Methanohalobium* (51.8%), *Harlarchaem* (14.7%), *Halolamina* (3.9%), *Halopenitus* (3.7%), *Halanaeroarchaeum* (3.1%), *Haloparvum* (2.9%), *Candidatus Haloredivivus* (2.2%), *Halobellus* (2.1%), and a genus (*Incertae Sedis*) of Nanohaloarchaeota (2.1%). Three of them were also indicator genera (*Candidatus Haloredividus*, *Halarchaeum*, and *Halolamina*). The rest of the archaeal genera represented 12.4%. Cluster C contained only one archaeal genus, which was an unassigned taxon within the phyllum Nanoarchaeota and was an indicator genus of this cluster. Two genera were mostly found in controls, which were unassigned taxa within phyla Iainarchaeota (93.2%) and Thermoplasmatota (3.0%). The indicator genera of each cluster are listed in Table S5.

The most abundant bacterial genera (> 2%) were also identified for each microbial cluster (Fig. 4b). Each cluster displayed abundant bacterial genera that were poorly represented in the other clusters. Cluster A was predominantly composed of *Pseudothermotoga* (22.0%), *Coprothermobacter* (14.4%), *Sporosalibacterium* (8.2%), *Petrotoga* (4.9%), *Acetomicrobium* (3.5%), *Thermovirga* (3.3%), *Syntrophus* (2.6%), *Marinilabilia* (2.6%) and *Tangfeifania* (2.3%). The other less abundant genera (< 2%) represented 36.3% of the bacterial population. Three dominant genera were also indicator genera (*Acetomicrobium*, *Petotroga* and *Thermovirga*). Cluster B was primarily composed of *Ralstonia* (12.9%), *Aliifodinibius* (6.5%), *Noviherbaspirillum* (6.5%), *Burkholderia-Caballeronia-Paraburkholderia* (5.9%), an unidentified genus of Balneolota (4.1%), *Variovorax* (3.3%), *Spomorusa* (3.0%), *Dyella* (3.0%) and *Curvibacter* (2.8%). No indicator genus has been identified for this cluster. All the other genera with an abundance inferior to 2% represented 52.0% of the total bacterial community. Cluster C included *Halomonas* (28.6%), *ML310M-34* (21.2%), *Wenzhouxiangella* (18.9%), *Rhodohalobacter* (9.4%), *Salibacter* (3.2%), *Glycocaulis* (2.8%), *Urania-1B-19* (2.4%), *Arhodomonas* (2.3%), and Candidatus Eremiobacetora (2.0%). Among these dominant genera, seven were also indicator genera (*Arhodomonas*, *Glycocaulis*, *ML310M-34*, *Rhodohalobacter*, *Salibacter* and *Wenzhouxiangella*). The other less abundant bacterial genera represented 9.2%. In cluster Controls, various dominant genera were found with *Aeromonas* (9.4%), *Oleidesulfovibrio* (5.9%), *Hydrogenophilus* (5.56%), *Cutibacterium* (4.4%), *Moheibacter* (4.2%), *Escherichia-Shigella* (3.2%), *Dolosigranulum* (2.3%), *Staphylococcus* (2.3%), *Streptococcus* (2.1%), *Geosporobacter* (2.0%) and *Dehalococcoides* (2.0%). The other less abundant genera represented 56.7% of the total bacterial community. All the indicator genera of each cluster are listed in Tables S6.

#### Bioinformatics Analysis of Hydrogen-consuming Organisms in Microbial Clusters

To assess whether metabolic activity could be associated with the microbial community structure, the bioinformatic tool “Tabigecy“ was applied to infer the community’s metabolic potential. This approach estimates metabolic functions based on the closest related taxa with known proteomes corresponding to those identified through 16S rRNA gene sequencing. In our case, most functional predictions were made at the Genus or Family level, suggesting a reasonably predictive capacity (Fig. S8). Distinct metabolic profiles were predicted for microbial clusters A, B and C (Fig. S9). Cluster A was characterized by a significantly higher median abundance of organisms capable of methanogenesis (7%), hydrogen generation (74%), and selenate reduction (30%) than clusters B and C, where these predicted functions were nearly absent or minimal (0–5%). In contrast, acetate oxidation potential was dominant in clusters B and C (74% and 85%, respectively), whereas it was limited to only 12% in Cluster A. Cluster C specifically showed high levels of acetogenesis (65%), methanotrophy (61%), and nitrate reduction (53%), a trend driven primarily by the prevalence of the *Sporomusa* genus. It is important to note that the detection of metabolic potential does not necessarily correspond to active metabolic processes in the sampled environments. For example, aerobic respiration was predicted in several taxa from salt caverns, despite these environments being anaerobic (Fig. S9). This unexpected finding may be explained in two ways: either the organisms utilize structurally and functionally similar cytochromes, markers for this function, to carry out respiration without oxygen as the final electron acceptor; or the aerobic respiration function was transferred from their closest known relatives, which inhabit oxygen-rich environments such as seawater, salt lakes, or sediments. The latter explanation is particularly relevant in understudied environments like salt caverns, where many microbial groups are still poorly characterized.

**Fig. 5:**
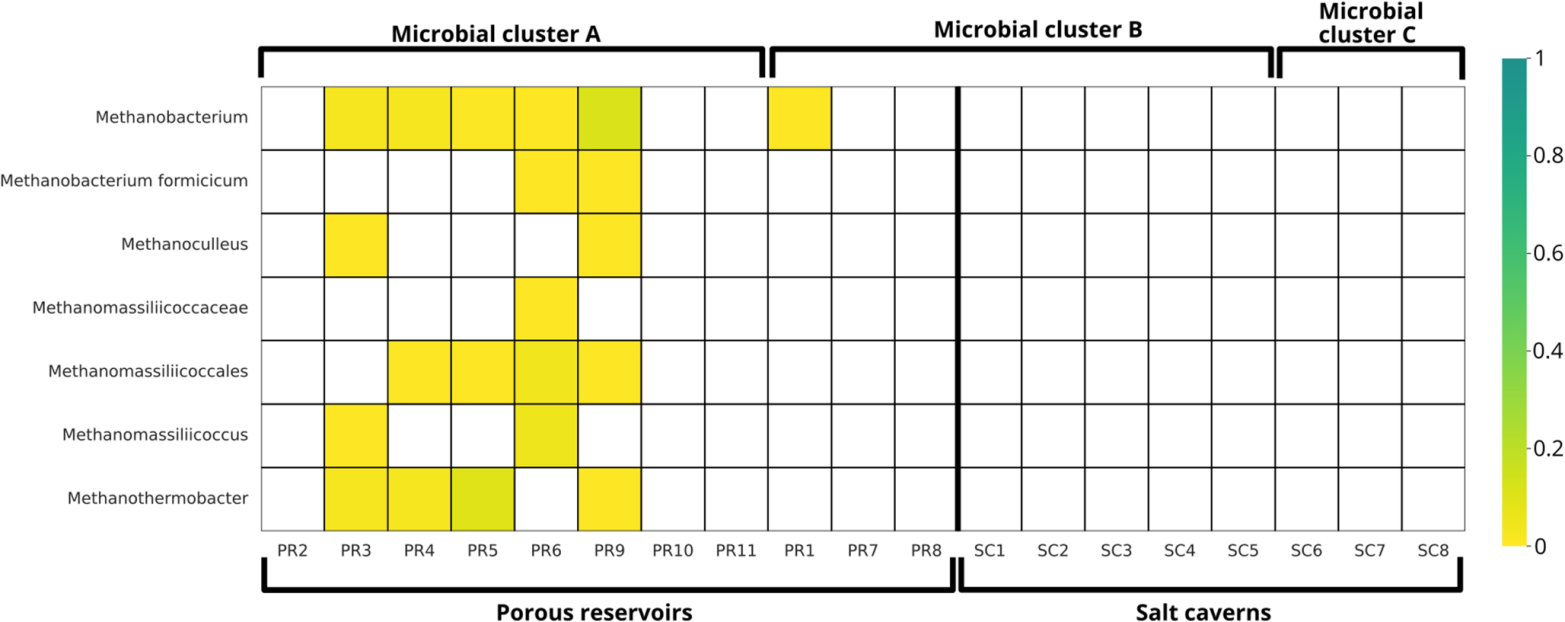
Abundance of taxa inferred to have hydrogen-consuming methanogenic potential based on 16S rRNA gene sequencing. Metabolic predictions were made using Tabigecy and filtered (see Methods) to identify taxon names (and all of the corresponding ASVs) with hydrogen-consuming methanogenic potential. Taxon names are associated with different taxonomic levels (genus, family, order), since some ASVs could not be identified precisely at species, genus or family levels. The abundance scale corresponds to the ratio obtained by dividing the sum of the abundances of ASVs associated with the taxon names by the total abundance of all ASVs in the sample. Samples names are sorted according to their microbial cluster identified in Fig. 3 and labelled by their reservoir type

Given the impact of methanogenesis on hydrogen levels, as well as the differences in this metabolic capacity observed among microbial clusters, our next analysis focused on hydrogen-consuming methanogens (Fig. 5). These organisms were identified in Cluster A (PR3-6 and PR9), which includes most porous reservoir samples, as well as in a single sample from Cluster B (PR1). The methanogenic communities in these samples comprise known methylotrophic and hydrogenotrophic taxa, including *Methanothermobacter*, *Methanobacterium*, and *Methanomassiliicoccus*, all of which use hydrogen as an electron donor [53, 54]. This suggests that hydrogen-consuming methanogenesis may occur in porous reservoirs. Dedicated growth experiments of these communities in the presence of hydrogen would be needed to validate this interpretation.

#### Impact of Environmental Stress and Human Activities on Microbial Communities

Some samples exhibited markedly different microorganism abundances, possibly due to environmental or human-induced stress. Genus *Halomonas* dominated samples SC6, SC7 and SC8 (53%, 66% and 44% of the total abundance, respectively) and was barely found in other samples. This could be explained by the alkalinity (pH > 11) of the brine induced by human activities (injection of NaOH), for which several species of *Halomonas* have shown adaptation [55]. Sample PR8 exhibited a high abundance of organisms belonging to the genus *Sporomusa*, with potential spore-forming functions (Fig S10). The stress induced by the high reservoir temperature (150 °C) or temperature variance in the reservoir has most likely exerted selective pressure on the community, favoring spore-forming organisms [56]. In summary, it can be seen that environmental and human-induced stress can exert a selective pressure on microbial communities.

### Chemical Control of Microbial Community Structure

#### Correlation between Chemical Characteristics and Microbial Assemblages

**Fig. 6:**
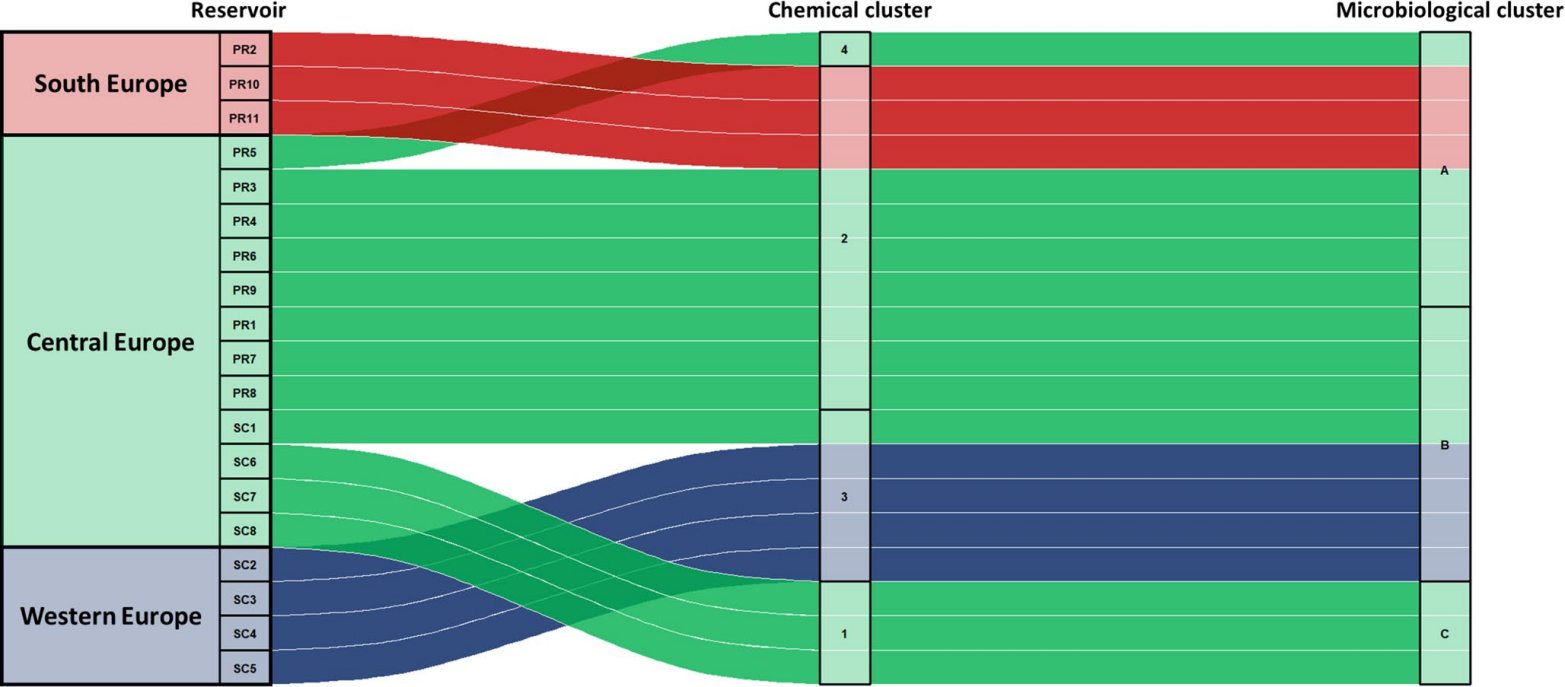
Association between the Europe areas, the chemical Cluster (determined by PCA) and the microbiology Cluster (determined by NMDS) for each reservoir sampled. The color indicates the area of origin with red for south Europe, green for central Europe and blue for western Europe

The correlations between the chemical characteristics, the chemical (determined by PCA) and microbiological (determined by NMDS) Clusters, and the origin area are illustrated in Fig. 6. Sites belonging to chemical Cluster 1, all located in central Europe (SC6, SC7 and SC8) were systematically associated with microbial Cluster C. Reservoirs classified in chemical Cluster 2 were divided into two microbial Clusters: Cluster A for sites in south Europe (PR2, PR10 and PR11) and some reservoirs in central Europe (PR3, PR4, PR5, PR6 and PR9), and Cluster B for sites in central Europe (PR1, PR7 and PR8). Sites associated with chemical Cluster 3, located in Western Europe, had microbial communities corresponding to Cluster B. Finally, the single site of chemical Cluster 4 (PR5), originating from central Europe, harbored a microbial community affiliated with Cluster A. These results underline that chemical facies constitute an important ecological factor in the structuring of microbial communities.

#### Functional Structuring of Archaeal and Bacterial Communities

**Fig. 7:**
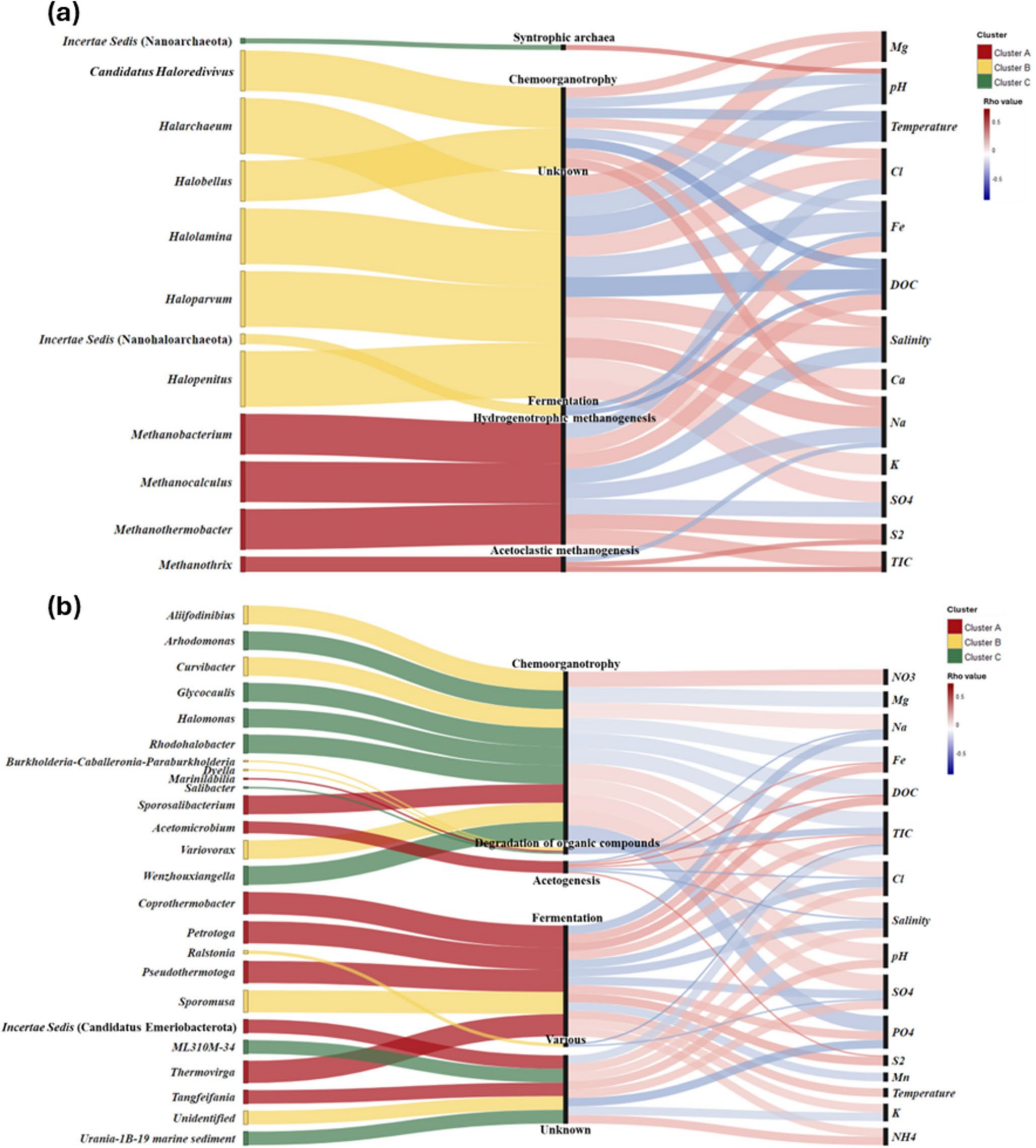
Sankey diagram representing a categorization of dominant archaea (**a**) and bacteria (**b**) according to their putative metabolism, associated with Spearman tests performed with physicochemical parameters. The putative metabolism of each genus has been attributed according the literature (see Table S7). Genus-function traits are colored according to the microbiological clusters determined: A in red, B in yellow, and C in green. Function-parameter lines are colored according to the *rho* value obtained for significant Mantel tests (*p* < 0.05). Red lines represent positive correlations and blue negative correlations. Complete results are indexed in Table S8 and Table S9

To study the ecology of microbial communities, the dominant archaeal and bacterial genera were classified into putative metabolic groups based on their functional characteristics described in the literature (Table S7) and then correlated with the physicochemical parameters of the reservoir fluids (Fig. 7). Archaeal communities showed a clear functional differentiation between clusters. Cluster A was dominated by acetoclastic (*Methanothrix*) and hydrogenotrophic methanogens (*Methanothermobacter*, *Methanocalculus*, *Methanobacterium*), whose relative abundance was positively correlated with DOC, DIC, Fe and S^2−^, indicating a dependence on carbon availability and reducing conditions. In contrast, the archaeal community of Cluster B were mainly composed of halophilic and chemoorganotrophic taxa (for example *Halarchaeum*, *Halobellus*, *Halolamina*), including several poorly characterized members of Halobacteriota, known to thrive in saline environments. These taxa were positively correlated with salinity indicators and negatively associated with carbon-related variables. The only dominant genus of Cluster C belongs to Nanoarchaeota, which could be a symbiont. Methylotrophic methanogens and sulfate-respiring archaea did not show significant environmental associations. The bacterial communities exhibited a comparable functional organization. In all samples, fermentation and chemoorganotrophy represented the dominant metabolic strategies. Cluster A was enriched in fermentative (for example *Thermovirga*, *Pseudothermotoga*) and acetogenic bacteria (*Acetomicrobium*), confirming the interpretation of carbon-dependent microbial activity in this type of reservoir. Conversely, the communities in Cluster B were largely dominated by chemoorganotrophic taxa (for example *Aliifonibius*, *Curvibacter*) adapted to saline conditions. Cluster C displayed a more heterogeneous functional composition, combining chemoorganotrophic bacteria (for example *Halomonas*, *Arhodomonas*), organic matter degraders (*Salibacter*), and taxa whose metabolic capacities remain poorly characterized, but with genera adapted to hypersaline conditions. Correlation analyses confirmed these ecological trends: fermentative and organic matter-degrading bacteria were positively associated with DOC, TIC, Fe, and S^2−^, while chemoorganotrophic and metabolically undefined taxa were linked to salinity indicators and NO_3_ concentrations.

To support the ecological interpretation, a redundancy analysis (RDA) was performed to identify the main environmental factors influencing the composition of microbial communities (Fig. S11). The environmental vectors revealed two major geochemical gradients. A salinity-related gradient, defined by increasing concentration of Cl and SO_4_, and another gradient associated with high concentrations of carbon and important temperatures. Samples of Cluster A are distributed along this last gradient and are characterized by anaerobic and carbon-associated taxa. Conversely, Cluster B are strongly aligned along the saline gradient, indicating selection favoring halophilic and halotolerant taxa. The samples in Cluster C follow a different way, with high pH and NH_4_ concentrations, and probably involve alkaliphilic microorganisms. Although the waters of PR5 are chemically distinct from those of other porous reservoirs (see PCA, Fig. 2a), the microbial community is organized along the same chemical gradients. In contrast, PR1, PR7, and PR8, which have a profile similar to other porous reservoirs, harbor microbial communities structured according to salinity gradients, like salt caverns.

## Discussion

Underground reservoirs are increasingly recognized as hosting microbial communities that may interact with injected H_2_. In this study, we provide a combined geochemical and microbiological characterization of 19 European reservoirs to evaluate factors potentially influencing H_2_ storage.

### Reliability and Representativeness of Microbial Sampling

The microbial communities identified in the studied reservoirs were primarily composed of taxa frequently encountered in anoxic subsurface ecosystems (for example: Bacteroidota, Desulfovibriona, Methanomicrobia) [16, 17, 57], including methanogenic archaea, fermentative bacteria and halophilic microorganisms [58–60]. Although contamination was present in the samples (for example: Chlamydiota) [61], a notable separation was observed between the environmental samples and the kit controls, confirming the authenticity of the microbial signatures detected in the samples. Consequently, the different sampling methods yielded reservoir or water column waters with a different signature than the controls.

### Influence of Water Chemical and Physical Parameters on Microbial Communities in Porous Reservoirs and Salt Caverns

The sampled underground gas reservoirs comprise two distinct geological settings: salt caverns (SC) and porous reservoirs (PR), each exhibiting distinct water chemistries and microbial communities (Table 1, Table S1 and Fig. S5). Salt caverns are characterized by high salinity and elevated SO₄^2−^ concentrations, whereas porous reservoirs display higher Fe and carbonate concentrations (Fig. 7). This geochemical contrast is mirrored in the microbial assemblages inhabiting these environments. Carbonate-rich porous reservoir (PR2 to PR6 and PR9 to PR11) communities seem to be dominated by acetoclastic and hydrogenotrophic methanogens, fermentative and acetogenic bacteria, typically associated with carbonate environments [62–64]. Although PR5 exhibits a distinct geochemical composition, with higher concentrations of carbon, potassium, and phosphate (Table S1), its microbial community remains similar to that of other porous reservoirs. This suggests that these parameters are not primary limiting factors in these environments, where community structure is more determined by redox conditions, salinity, the availability of reduced substrates, and a minimum required carbon concentration. Metabolically, these microbial communities seem to be oriented toward H_2_ production and consumption. The chemical conditions prevailing in these reservoirs, including reducing conditions and the presence of residual hydrocarbons, promote the generation of H_2_ and small organic compounds, which in turn support methanogenesis [65–67]. The co-occurrence of methanogens and fermentative bacteria reflects well-established anaerobic degradation pathways in carbonate-rich reducing subsurface environments, often under elevated temperature conditions [68, 69]. Organic carbon availability is a typically critical limiting factor in deep anaerobic ecosystems [70–72].

In contrast, microbial communities in salt caverns (SC1 to SC8) and certain porous reservoirs (PR1, PR7 and PR8) seem to be dominated by halophilic and generalist taxa not involved in methanogenesis (Fig. 7). Saline environments are well known to favor halophilic microorganisms [58]. These environments, rich in sodium and chloride, exert a high osmotic pressure that selects for microorganisms capable of specific adaptations to maintain their osmotic balance [73, 74]. As a result, energy-intensive metabolic pathways like methanogenesis are less likely under these conditions. Interestingly, PR7, classified as a high-salinity porous reservoir, shares both microbial and chemical profiles with salt caverns [75]. Even more puzzling are the microbial communities in PR1 and PR8, which resemble those of salt caverns despite their low salinity and low SO_₄_^²⁻^ concentrations, typical of other porous reservoirs. Two hypotheses may explain this observation: (i) these reservoirs could have inherited ancient brines, allowing halophiles to persist in an oligotrophic environment due to low competition, or (ii) local saline micro-niches not detected by general chemical analyses could allow the persistence of these halophiles [75, 76]. Similar to observations in deep fracture fluids of the Kidd Creek Observatory, halophilic taxa can persist in anoxic, deep subsurface environments that are not strictly hypersaline. In these systems, limited but persistent sources of carbon from ancient rock layers support the survival and activity of halophilic microorganisms [77]. Dutta et al. (2019) demonstrated the co-presence of halophilic archaea with acidophilic and methanogenic archaea in oligotrophic crustal systems, highlighting that local geological niches can allow the coexistence of diverse physiological groups [78]. In another study, Gales et al. (2016) demonstrated the presence of aerobic microorganisms in a strictly anaerobic and saline environment, suggesting that the microorganisms are either inactive and their DNA has been preserved for many years, or that microbial activity is low and the community has maintained itself in a state of slow growth [79]. All these studies suggest that the identified microbial communities do not solely represent active microorganisms, but that the microbial signature reflects the history of the underground.

Beyond water chemistry, geographic origin appears to influence microbial community composition. For instance, reservoirs from central and south Europe, which are typical of carbonate-rich waters, are largely dominated by methanogenic taxa (Figs. 4, 5 and 6). On the other hand, reservoirs from western Europe, and some reservoirs of central Europe, exhibited higher relative abundances of halophilic taxa, both in salt caverns and porous reservoir samples. These trends suggest that geological factors significantly shape the structure of deep subsurface biospheres. Although the results are consistent with previous studies showing that substrate mineralogy, porosity and water-rock interactions are critical determinants of microbial community structure in the deep subsurface [80–83], further data acquisition is needed to confirm these observations.

### Impacts of Anthropogenic Activities on Microbial Communities and Potential Metabolisms

Although most microbial community profiles align with the natural geochemical and geological reservoir characteristics, several of them exhibit atypical features potentially resulting from anthropogenic interventions. Reservoirs subjected to industrial operations show altered subsurface conditions and shifts in microbial community composition. For instance, SC6, SC7, and SC8 present extremely alkaline pH values (> 11), which are not typical of natural deep subsurface environments. This alkalinity is attributable to the use of alkaline anticorrosive treatments in salt caverns [84–86]. These harsh chemical conditions significantly reduce microbial diversity and select for a narrow range of alkaliphilic taxa, including *Halomonas*, which dominates the microbial profile of these samples. The genus *Halomonas* has also been found in hypersaline environments, such as the Jintan mine [87] and a warm saline formation porewater from the Cambrian Mt. Simon Sandstone [88]. Contaminations with specific chemicals are known to trigger a decrease in overall microbial diversity while increasing specific functional groups, often consisting of microorganisms resistant to altered conditions [89, 90]. This simplification of microbial networks suggests reduced ecological resilience and a possible shift in metabolic pathways toward generalist, stress-resistant functions, reflecting a specific adaptation to the altered pH. Similarly, PR5 underwent accidental lubricant injection, introducing a complex anthropogenic source of organic matter while also increasing the pH. This perturbation resulted in altered water geochemistry, with elevated phosphate, sulfide, and potassium concentrations. These anthropogenic changes appear to promote the proliferation of spore-forming *Firmicutes* in several samples, known for their ability to produce resistant endospores under stress conditions [91, 92]. However, spore-forming Firmicutes are naturally adapted to survive in deep-water environments [93, 94]. While these organisms may not be metabolically active, their persistence in dormant form contributes to a microbial “seed bank” adapted to environmental disturbances. The detection of such sporulating genera confirms the exposure of PR5 to significant anthropogenic stress. In contrast to these observations, salt cavern SC3, which is used for ethylene storage, shows a similar chemistry of the brine and microbial communities compared to other salt caverns and does not appear to be affected by the chemical. This could be due to the low bioavailability or low concentration of ethylene in the aqueous phase, the absence of ethylene-using microorganisms among the resident communities, or the major constraints imposed by high salinity that govern microbial activity in this environment. Together, these examples underscore that industrial activities can substantially and long lastingly reshape microbial community composition. While the core functional potential of the communities may be maintained, their structure, diversity, and ecological resilience are clearly impacted. These findings highlight the necessity of incorporating operational history when interpreting microbial data from subsurface environments, especially in the context of environmental monitoring or biotechnological applications. In the same way, any human action on a reservoir has to be carefully considered.

### Taxa as Indicators

The composition of microbial communities in the studied subsurface environments reveals specific taxa whose occurrence or relative abundance is strongly associated with particular reservoir types or geochemical conditions. In porous reservoirs, several microbial taxa appear to be characteristic for carbonate-rich anaerobic conditions [95]. Methanogenic archaea such as *Methanothermobacter*, *Methanobacterium*, *Methanothrix*, and *Methanocalculus*, along acetogenic bacteria, such as *Acetomicrobium*, are recurrently enriched in these environments. Their co-occurrence indicates the presence of robust syntrophic networks supporting hydrogenotrophic and acetoclastic methanogenesis, metabolic pathways commonly favored in reducing environments with elevated organic carbon availability, likely derived from residual hydrocarbons and their degradation products [96, 97]. Additionally, the frequent detection of thermophilic genera such as *Thermovirga* and *Petrotoga* in high-temperature reservoirs underscores their role as reliable indicators of deep, warm, and thermally active anaerobic niches. In contrast, microbial communities in salt caverns are dominated by halophilic archaea such as *Halarchaeum*,* Haloarcula*,* Halanaeroarchaeum*, or *Halobellys*. These taxa form a characteristic consortium that serves as an effective biological signature of hypersaline conditions [58, 98]. Their recurrent presence across multiple salt cavern samples reinforces their reliability as indicators of saline geochemical regimes. Collectively, these indicator taxa provide a biological readout of both geochemical states and anthropogenic disturbances in subsurface environments and potential hydrogen storage sites. Their presence, relative abundance, or absence can inform reconstructions of reservoir history, evaluations of ecological resilience, and assessments of microbial risks to technical systems, including those related to hydrogen storage integrity and biocorrosion.

### Considerations for Hydrogen Storage: Risks of Microbial Consumption

The deep subsurface environments investigated in this study provide critical insights into microbial processes that may influence long-term hydrogen storage. While many reservoirs appear to harbor communities with potentially limited metabolic activity due to harsh physical or chemical conditions, several factors suggest that microbial hydrogen consumption represents a potential risk, particularly in porous reservoirs. Carbonate-rich porous reservoirs enriched in methanogenic archaea and hydrogenotrophic syntrophic bacteria, especially those with functional signatures of active hydrogenase genes (involved in methanogenesis or acetogenesis), are of particular concern. These consortia are known to be capable of hydrogenotrophic methanogenesis, wherein H_2_ is consumed to reduce CO_2_ to CH_4_. This metabolic activity could result in both the loss of stored hydrogen and the unintended production of methane. Additionally, the presence of hydrogen-producing fermentation pathways in these communities indicates a microbial network already structured around hydrogen cycling. In the context of underground hydrogen storage, such microbial communities can contribute to significant hydrogen consumption, with methanogenesis [36], sulfate reduction or homoacetogenesis [99]. While this does not preclude storage feasibility, it highlights the need for further investigation, particularly through growth and kinetic experiments at various scales, to better quantify microbial impacts and inform operational strategies. In addition, metabolic signatures related to acetogenesis and sulfate reduction, both of which are likely to consume hydrogen, have also been identified in the microbial communities of salt caverns.

It is clear that a limitation of the performed analyses is the primary focus on dominant taxa in the liquid brine, meaning that potentially active hydrogen-consuming organisms associated with rock surfaces or biofilms may remain undetected. It is well known that microbial communities inhabiting solid matrices can differ substantially from those found in the aqueous phase [89, 100]. Biofilms can up-concentrate microbial cells and facilitate metabolic interactions, potentially enhancing hydrogen consumption compared to the planktonic phase. In addition, biofilm growth and microbially induced mineral precipitation could alter the physical properties of the reservoirs, such as porosity and permeability, and thereby influence gas diffusion and storage efficiency. As such, the functional inferences derived from water-phase microbial data and geochemistry likely capture only a portion of the present groups. It should also be emphasized that the present study focuses on a limited number of reservoirs. Therefore, any generalization to all potential hydrogen storage sites should be made with caution. To validate and refine the trends observed in this study, investigations are underway to expand the dataset by including additional reservoirs.

All our findings are based on 16 S rRNA gene sequencing and predictive functional analyses. While this approach provides a comprehensive overview of community composition across numerous reservoirs, it does not distinguish between metabolically active and dormant organisms. Given that all DNA is sequenced regardless of metabolic state, the actual activity of the microbial community may be over- or underestimated. To assess the real impact of microbial communities on hydrogen storage, complementary investigations are required. Future studies should include enrichment cultures to identify H_2_-consuming organisms under reservoir-relevant conditions, as well as metagenome-assembled genomes and meta-transcriptomics to directly assess the potential for hydrogen metabolism. In this context, understanding the baseline microbiological potential of each reservoir is essential before considering its use for hydrogen storage. Monitoring key indicator taxa, evaluating metabolic gene content, and tracking community shifts during pilot injection trials can help anticipate microbial risks. Such knowledge can inform mitigation strategies, including reservoir selection, monitoring protocols, and targeted biocide application, ultimately supporting the safe and efficient deployment of underground hydrogen storage technologies.

## Conclusion

This study provides a characterization of the geochemical and microbiological diversity of various deep salt caverns and porous reservoirs in Europe, with a focus on their potential for underground H_2_ storage use. Different reservoir fluid sampling methods were used to create an inventory of the microbial communities present. The results show that, although water chemical characteristics (i.e. pH, salinity, carbon availability) influence microbial community structure, other environmental factors such as temperature, geology, and anthropogenic activities can also modify it. A clear distinction between salt caverns and porous reservoirs was observed, where porous reservoirs, which are rich in carbonates, harbor microbial communities that promote methanogenesis and acetogenesis. Saline reservoirs are dominated by halophilic communities but also carry potential H_2_-consuming microorganisms. The reservoirs with substantial anthropogenic impact highlight the selection of specific taxa under extreme conditions, while also highlighting the limitations of 16 S sequencing for assessing active microbial processes in situ, particularly under conditions where microbial activity is unlikely. Given the prevalence of microbial groups capable of potentially consuming H_2_ in carbonate and saline reservoirs, it is important to go beyond detection and assess their actual activity. Future work should therefore also focus on assessing the actual H_2_ consumption potential of these microbial communities. In conclusion, understanding the interaction between reservoir chemistry, geology, and microbiology is essential to ensure better control of H_2_ storage processes in the context of energy transitions.

## Supplementary Information

Below is the link to the electronic supplementary material.

Supplementary Material 1

## Data Availability

The data supporting this article has been included as part of the Supplementary Information. Sequencing data have been deposited in the NCBI Sequence Read Archive under BioProject accession number [PRJNA1328997] (https:/www.ncbi.nlm.nih.gov/bioproject/PRJNA1328997).
