# Supplementary material for: Microbial-geochemical Interactions in Underground Reservoirs: Implications for Hydrogen Storage
Source: Microb Ecol. 2026 Jun 5;89:156. doi: 10.1007/s00248-026-02800-8 (PMC13461944; doi:10.1007/s00248-026-02800-8)
Supplement: Supplementary file 1 [file 248_2026_2800_MOESM1_ESM.docx]

**Supporting Information**

**Microbial-geochemical interactions in underground reservoirs: Implications for hydrogen storage**

Laura Fablet^1^, Arnaud Belcour^2,3^, Sylvain Stephant^1^, Caroline Michel^1^, Delphine Ropers^2,3^, Katerina Cerna^5^, Petra Bombach^6^, Jakub Riha^5^, Joachim Tremosa^7^, Biwen Annie An-Stepec^4^, Kristyna Fadrhonc^5^, Nicole Dopffel^4^, Setareh Rad^1^

^1^ BRGM, 3 Avenue Claude Guillemin, 45060 Orléans Cedex 2, France

^2^ Univ. Grenoble Alpes, Inria, 38000 Grenoble, France

^3^ Univ. Grenoble Alpes, CNRS, LIPhy, Grenoble, France

^4^ NORCE Research AS, Nygardsgaten 112, 5008 Bergen, Norway

^5^Technical University of Liberec, Institute for Nanomaterials, Advanced Technologies and Innovation, Bendlova 7, Liberec, Czechia

^6^ Isodetect GmbH, Deutscher Platz 5b, 04103 Leipzig, Germany

^7^ Geostock, 2 rue des Martinets, 92500 Rueil-Malmaison, France

**The supporting information section contains 29 pages, 58 reference, 11 figures and 9 tables.**

**Table S1**	Physico-chemical parameters of groundwater samples providing of porous reservoirs (PR) and salt caverns (SC) (n.a. = not-analyzed).

| **Parameters** | **PR1** | **PR2** | **PR3** | **PR4** | **PR5** | **PR6** | **PR7** | **PR8** | **PR9** | **PR10** | **PR11** |
| --- | --- | --- | --- | --- | --- | --- | --- | --- | --- | --- | --- |
| **SO_4_ (mg/L)** | 0.48 | 1035 | 12 | <5 | 2970 | 50 | <0.5 | <0.5 | 3 | 5.8 | 0.1 |
| **Cl (mg/L)** | 13230 | 6130 | 6000 | 9212 | 13750 | 1020 | 110000 | 40 | 10100 | 55803 | 549000 |
| **PO_4_ (mg/L)** | <0.1 | 0 | 0.1 | 1.8 | 47.5 | 0.3 | <0.01 | <0.01 | 0.1 | n.a | 0.1 |
| **NO_3_ (mg/L)** | <0.1 | 0 | 6 | <5 | 5 | 10 | 630 | <0.01 | 0.1 | n.a | 0.1 |
| **NO_2_ (mg/L)** | <0.1 | 0 | 15.1 | 0.2 | 14.5 | 5.7 | <0.02 | <0.01 | 0.1 | n.a | 0.1 |
| **S_2_ (mg/L)** | n.a | <1 | <1 | 0.2 | 13.7 | 0.1 | <0.05 | <0.05 | n.a | 0 | 0 |
| **Na (mg/L)** | 6565 | 4610 | 3550 | 6474 | 3650 | 996 | 21300 | 1.59 | 6075 | 25300 | 25300 |
| **Ca (mg/L)** | n.a | 830 | 123 | <50 | 48 | 14.3 | 42500 | 12.9 | 287.5 | 3919 | 3725 |
| **Mg (mg/L)** | n.a | 160 | 200 | <50 | 11.4 | 4.8 | 86.2 | 0.3 | 175 | 1535 | 1515 |
| **Mn (mg/L)** | n.a | n.a | n.a | 0.02 | n.a | 0.26 | 48 | 0.07 | n.a | n.a | n.a |
| **K (mg/L)** | 700 | 115 | 1465 | 410 | 52200 | 11.3 | 1730 | 1.87 | 132.5 | 412 | 402 |
| **NH_4_ (mg/L)** | <0.1 | n.a | n.a | 66.4 | n.a | 6.9 | 1.71 | 4.9 | n.a | n.a | n.a |
| **Fe (mg/L)** | 35.9 | 1.3 | 19.5 | 73.2 | 10.75 | 7.25 | 11.2 | 0.45 | 0.9 | 25 | 31 |
| **HCO_3_ (mg/L)** | n.a | 164.9 | 1166 | 2452 | 25945 | 1172 | 2.8 | 7.9 | 191.4 | 22.9 | 5 |
| **CO_3_ (mg/L)** | n.a | 0.6 | 2.52 | 18.43 | 9882 | 17.06 | 0.001 | 0.011 | 0.65 | 0.01 | 0.002 |
| **TIC (mg/L)** | n.a | 38.7 | 272.6 | 563 | 7212 | 244 | 2.1 | 1.7 | 44.4 | 8.9 | 2.2 |
| **DOC (mg/L)** | n.a | 598 | 445.6 | 127 | 2088.9 | 20 | 22.8 | 12.9 | 96.2 | 40.7 | 469.3 |
| **pH** | 8.1 | 7.4 | 7.2 | 7.7 | 9.2 | 8.2 | 5.9 | 7.4 | 7.4 | 6.3 | 6.1 |
| **Eh (mV)** | n.a | 232 | -15 | -57 | -300 | -172 | n.a | n.a | -108 | n.a | n.a |
| **Salinity (g/L)** | 20.53 | 13 | 11.71 | 16.86 | 79.94 | 2.39 | 176.31 | 0.06 | 10.83 | 87 | 85.87 |
| **Temperature (°C)** | 13.3 | 90 | 49 | 47 | 44 | 41 | 150 | 150 | 39 | 61.5 | 61.5 |
| **Parameters** | **SC1** | **SC2** | **SC3** | **SC4** | **SC5** | **SC6** | **SC7** | **SC8** |  |  |  |
| **SO_4_ (mg/L)** | 4644 | 3891 | 3990 | 3590 | 11200 | 5200 | 4550 | 4650 |  |  |  |
| **Cl (mg/L)** | 197303 | 184564 | 182379 | 196500 | 244000 | 194000 | 188000 | 184000 |  |  |  |
| **PO_4_ (mg/L)** | <1 | <0.1 | <0.1 | <0.04 | <0.04 | <250 | <250 | <250 |  |  |  |
| **NO_3_ (mg/L)** | n.a | <50 | <50 | <250 | <250 | <250 | <250 | <250 |  |  |  |
| **NO_2_ (mg/L)** | n.a | <0.01 | <0.01 | <250 | <250 | <250 | <250 | <250 |  |  |  |
| **S_2_ (mg/L)** | <1 | <0.1 | <0.1 | <0.05 | <0.05 | n.a | n.a | n.a |  |  |  |
| **Na (mg/L)** | 129313 | 116190 | 114326 | 114000 | 146000 | 109000 | 114000 | 114000 |  |  |  |
| **Ca (mg/L)** | n.a | 1522 | 1499 | 1870 | 1540 | 26.1 | 20.2 | 114 |  |  |  |
| **Mg (mg/L)** | n.a | 489 | 448 | 1305 | 383 | 3.3 | 2 | 2.5 |  |  |  |
| **Mn (mg/L)** | n.a | 0.11 | 0.13 | 3.54 | 0.18 | 0.31 | 0.2 | 0.23 |  |  |  |
| **K (mg/L)** | 42.5 | 662 | 646 | 1220 | 1260 | 66.8 | 105 | 76.6 |  |  |  |
| **NH_4_ (mg/L)** | <1 | 3.2 | 2.4 | 8.5 | 2.1 | 8.1 | 118 | 6.3 |  |  |  |
| **Fe (mg/L)** | 0.35 | 0 | <20 | 8.58 | 3.04 | 1.27 | 0.67 | 0.85 |  |  |  |
| **HCO_3_ (mg/L)** | 159.4 | 16.7 | 11.3 | 63.5 | 12.7 | n.a | n.a | n.a |  |  |  |
| **CO_3_ (mg/L)** | 0.19 | 0.03 | 0 | 0.13 | 0.02 | n.a | n.a | n.a |  |  |  |
| **TIC (mg/L)** | 147 | 12.6 | 16 | 49 | 19 | n.a | n.a | n.a |  |  |  |
| **DOC (mg/L)** | n.a | 0.9 | 0.8 | 4.6 | 12.8 | n.a | n.a | n.a |  |  |  |
| **pH** | 6.6 | 6.8 | 5.1 | 6.8 | 6.7 | 11.4 | 11.5 | 11.4 |  |  |  |
| **Eh (mV)** | n.a | 246 | 26 | 264 | -90 | n.a | n.a | n.a |  |  |  |
| **Salinity (g/L)** | 331.45 | 307.48 | 303.45 | 318.91 | 404.41 | 308.31 | 306.8 | 302.85 |  |  |  |
| **Temperature (°C)** | 48 | 18 | 18 | 14.96 | 21 | 30 | 30 | 30 |  |  |  |

**Table S2**	Heatmap showing the strength of the correlation between physicochemical parameters and principal component (PC) axis. Significant variables (p < 0.05) are shown in bold. The color varies from red to green depending on the intensity of the correlation strength.

| **Physicochemical parameters** | **Dim.1** | **Dim.2** |
| --- | --- | --- |
| **SO_4_** | 0.02 | **0.83** |
| **Cl** | 0.2 | **0.72** |
| **PO_4_** | **0.97** | 0.03 |
| **S_2_** | **0.96** | 0.03 |
| **Na** | 0.2 | **0.75** |
| **Ca** | 0.01 | 0.04 |
| **Mg** | 0.05 | 0.01 |
| **Mn** | 0.04 | 0.05 |
| **K** | **0.92** | 0.05 |
| **NH_4_** | **0.89** | 0.03 |
| **Fe** | 0.01 | **0.26** |
| **HCO_3_** | **0.96** | 0.03 |
| **CO_3_** | **0.95** | 0.03 |
| **TIC** | **0.96** | 0.03 |
| **DOC** | 0.86 | 0.02 |
| **pH** | 0.02 | 0.15 |
| **Salinity** | 0.14 | **0.78** |
| **Eh** | 0.07 | 0.12 |
| **Temperature** | 0.01 | **0.03** |

 **Fig. S1** Principal Component Analyses (PCA) biplot of groundwater samples based on their chemical composition and regrouped in four clusters. The principal components 2 and 3, (Dim.2 and Dim.3), explain 23.1% and 16.0% of the total variance, respectively. Arrows indicate the direction and strength of each chemical variable contribution to the principal components. Cluster 1 is represented by purple circles, Cluster 2 by green triangles, Cluster 3 by red squares and Cluster 4 by orange cross.

**Fig. S2**	Boxplots of the average concentrations of physico-chemical parameters by cluster. The p-value (*p*) obtained for the Kruskal-Wallis tests is indicated for each parameter. Significance stars displayed when the p-value is less than 0.05 represent significant differences between two groups according to Dunn’s test. It should be noted that small sample sizes in some clusters may reduce the sensitivity of Dunn’s test.

**Fig. S3**	Principal Component Analyses (PCA) biplot performed separately on the PR (a, b) and SC (c, d) samples. Arrows indicate the direction and strength of each chemical variable contribution to the principal components.

**Table S3**	Replicates of each sample for sequencing with Bray-Curtis distance calculated between replicates. SC = Salt cavern and PR = Porous reservoir.

| **Sample** | **Replicate 1** | **Replicate 2** | **Bray-Curtis distance** |
| --- | --- | --- | --- |
| PR1 | PR1.1 | PR1.2 | 0.353 |
| PR2 | PR2 |  |  |
| PR3 | PR3.1 | PR3.2 | 0.149 |
| PR4 | PR4.1 | PR4.2 | 0.0717 |
| PR5 | PR5.1 | PR5.2 | 0.0843 |
| PR6 | PR6.1 | PR6.2 | 0.07 |
| PR7 | PR7.1 | PR7.2 | 0.0471 |
| PR8 | PR8.1 | PR8.2 | 0.0887 |
| PR9 | PR9.1 | PR9.2 | 0.317 |
| PR10 | PR10.1 | PR10.2 | 0.0647 |
| PR11 | PR11.1 | PR11.2 | 0.053 |
| SC1 | SC1 |  |  |
| SC2 | SC2 |  |  |
| SC3 | SC3 |  |  |
| SC4 | SC4 |  |  |
| SC5 | SC5.1 | SC5.2 | 0.0568 |
| SC6 | SC6.1 | SC6.2 | 0.0503 |
| SC7 | SC7.1 | SC7.2 | 0.208 |
| SC8 | SC8.1 | SC8.2 | 0.113 |

**Fig. S4**	Non-metric multidimensional scaling (NMDS) plot showing the spatial distribution of each replicate, according to the calculated Bray-Curtis distance between replicates of Bacteria and Archaea sequencing data. A significant difference between groups was observed (Wilcoxon test, *p* < 0.001).

**Figure S5**	Relative abundance of microbial classes for each sampled porous reservoir (PR) and salt cavern (SC), as well as for the controls (C). In the legend, classes circled in black are at least common between two sample types, while those circled in green are unique to the PR, those in blue to the SC, and those in red to the controls.

**Fig. S6**	Alpha diversity indices: (a) specific richness, (b) Shannon index and (c) Simpson index, of clusters and controls for archaea and bacteria. *“p”* values are from Kruskal-Wallis test. The letters a and b are used to clarify whether the difference between any pair of groups calculated by Dunn’s test was statistically significant (p < 0.05), and there was a significant difference in flora diversity between the two groups sharing no common letter markers.

**Table S4**	Genera identified as potential contaminant using the decontamination prevalence method, with their presence/absence in samples

| **Identified contaminants** | | **Number of contaminated sites** | **Site concerned** |
| --- | --- | --- | --- |
| **Genus** | **Phylum** |  |  |
| *Atopostipes* | Bacillota | 0 |  |
| *Blastocatella* | Acidobacteriota | 0 |  |
| *Brevibacterium* | Actinomycetota | 0 |  |
| *Dolosigranulum* | Bacillota | 0 |  |
| *Escherichia-Shigella* | Pseudomonadota | 8 | PR1, PR4, PR7, PR8, PR9, PR11, SC2, SC3 |
| *Paludibaculum* | Acidobacteriota | 0 |  |
| *Pseudoalteromonas* | Pseudomonadota | 0 |  |
| *Rhodococcus* | Actinomycetota | 0 |  |
| *Rubellimicrobium* | Pseudomonadota | 0 |  |
| *Rubrobacter* | Actinomycetota | 0 |  |
| *SH-PL14* | Planctomycetota | 0 |  |

**Fig. S7**	Non-metric multidimensional scaling (NMDS) plot showing microbial community for (a) salt carverns (SC) and (b) porous reservoirs (PR) with the groups defined by clustering.

**Table S5**	List of indicator archaeal genera, which correspond to genera specifically associated with particular environmental conditions, for each cluster (A, B, C and Controls). Indicator taxa were identified using indicator species analysis. Only taxa with p-values < 0.01 were considered significant. The indicator genera were determined by the calcul of the indicator value, which corresponds to the probability that a genus is both frequent and faithful to a given group and varies between 0 and 1.

| **Genus** | **Phylum** | **Associated cluster** |
| --- | --- | --- |
| *Candidatus Haloredivivus* | Nanohaloarchaeota | Cluster B |
| *Halarchaeum* | Halobacteriota | Cluster B |
| *Halocarcula* | Halobacteriota | Cluster B |
| *Halolamina* | Halobacteriota | Cluster B |
| *Halomicrobium* | Halobacteriota | Cluster B |
| *Haloplanus* | Halobacteriota | Cluster B |
| *Incertae Sedis* | Halobacteriota | Cluster C |
| *Methanobacterium* | Methanobacteriota | Cluster A |
| *Methanocalculus* | Halobacteriota | Cluster A |
| *Methanothermobacter* | Methanothermobacteria | Cluster A |
| *Methanothrix* | Halobacteriota | Cluster A |
| *Natromonas* | Halobacteriota | Cluster B |
| *Unidentified* | Halobacteriota | Cluster B |

**Table S6**	List of indicator bacterial genera, which correspond to genera specifically associated with particular environmental conditions, for each cluster (A, B, C and Controls). Indicator taxa were identified using indicator species analysis. Only taxa with p-values < 0.05 were considered significant, to reduce false positives. The indicator genera were determined by the calcul of the indicator value, which corresponds to the probability that a genus is both frequent and faithful to a given group and varies between 0 and 1.

| **Genus** | **Phylum** | **Associated cluster** |
| --- | --- | --- |
| *Acetobacterium* | Bacillota | Cluster A |
| *Acetomicrobium* | Synergistota | Cluster A |
| *Arhodomonas* | Pseudomonadota | Cluster C |
| *Blastopirellula* | Planctomycetota | Cluster C |
| *Candidatus_Caldatribacterium* | Atribacterota | Cluster A |
| *Candidatus_Finniella* | Pseudomonadota | Cluster C |
| *Coxiella* | Pseudomonadota | Cluster C |
| *CSP1-2* | Dadabacteria | Cluster C |
| *Desulfitibacter* | Bacillota | Cluster A |
| *Gelria* | Bacillota | Cluster A |
| *Glycocaulis* | Pseudomonadota | Cluster C |
| hgcI_clade | Actinomycetota | Cluster C |
| *Hoppeia* | Bacteroidota | Cluster C |
| *Incertae_Sedis* | Chlamydiota | Cluster B |
| *Incertae_Sedis* | Dependentiae | Cluster B |
| *Incertae_Sedis* | Candidatus_Eremiobacterota | Cluster C |
| *JGI-0000079-D21* | Synergistota | Cluster A |
| *LD29* | Verrucomicrobiota | Cluster C |
| *ML310M-34* | Balneolota | Cluster C |
| *Moorella* | Bacillota | Cluster A |
| *Mycobacterium* | Actinomycetota | Cluster C |
| *Owenweeksia* | Bacteroidota | Cluster C |
| *Pelagibacterium* | Pseudomonadota | Cluster C |
| *Petrotoga* | Thermotogota | Cluster A |
| *Pirellula* | Planctomycetota | Cluster C |
| *Proteiniphilum* | Bacteroidota | Cluster A |
| *Rhodohalobacter* | Balneolota | Cluster C |
| *Salibacter* | Bacteroidota | Cluster C |
| *Salinarimonas* | Pseudomonadota | Cluster C |
| *Sumerlaea* | Sumerlaeota | Cluster C |
| *Thermodesulfovibrio* | Nitrospirota | Cluster A |
| *Thermovirga* | Synergistota | Cluster A |
| *Truepera* | Deinococcota | Cluster C |
| *Urania-1B-19_marine_sediment_group* | Planctomycetota | Cluster C |
| *Wenzhouxiangella* | Pseudomonadota | Cluster C |

**Fig. S8**	Barplot showing the taxonomic rank used by EsMeCaTa to make functional predictions with the relative abundance of the associated organisms for the samples.

**Fig. S9**	Heatmap indicating for each major metabolic function the relative abundance of microorganisms found in samples. Size and color of each bubble correspond to the relative abundance of the organisms predicted to possess these functions.

**Fig. 10**	Boxplot showing the relative abundance of organisms predicted to carry the sporulation gene *spo0A* across chemical clusters. Sample IDs are displayed next to their corresponding nodes.

**Table S7**	Natural habitat and main metabolism of dominant archaeal and bacterial genera, with the accorded phylum. For taxa that are still poorly understood, the habitat and/or metabolism may be considered unknown.

| **Genus** | **Phylum** | **Natural habitat** | **Main metabolism** |
| --- | --- | --- | --- |
| **Archaea** | | | |
| *Candidatus Haloredivivus* | Nanohaloarchaeota | Anaerobic hypersaline environments (i.e. salt lakes) [1] | Chemoorganotrophy [1] |
| *Candidatus Methanoplasma* | Thermoplasmatota | Thermophilic environments [2] | Methylotrophic methanogenesis [2] |
| *Halanaeroarchaeum* | Halobacteriota | Anaerobic hypersaline environments (i.e. salt lakes) [3] | Sulfur respiration [3] |
| *Halarchaeum* | Halobacteriota | Saline environments (i.e. solar salt) [4] | Unknown* |
| *Halobellus* | Halobacteriota | Aerobic hypersaline environments (i.e. salt lakes, marine solar salterns, subterranean salt deposits) [5] | Chemoorganotrophy [5] |
| *Halolamina* | Halobacteriota | Aerobic hypersaline environments (i.e. marine solar saltern) [6] | Unknown* |
| *Haloparvum* | Halobacteriota | Aerobic hypersaline environments [7] | Unknown* |
| *Halopenitus* | Halobacteriota | Hypersaline environment (i.e. salt lakes) [8] | Unknown* |
| *Incertae Sedis* | Nanohaloarchaeota | Hypersaline environments (i.e. solar salterns, salt lakes, deep-seau brine pools) [9] | Fermentation [10] |
| *Incertae Sedis* | Nanoarchaeota | Marine environments [11] | Symbiotic archaea [12] |
| *Methanobacterium* | Methanobacteriota | Anaerobic environments (i.e. wetlands, marshes, freshwater lakes) [13] | Hydrogenotrophic methanogenesis [13] |
| *Methanocalculus* | Halobacteriota | Anaerobic saline environments (i.e. oifield, hypersaline lakes) [14] | Hydrogenotrophic methanogenesis [15] |
| *Methanohalobium* | Halobacteriota | Anaerobic saline environments [16] | Methylotrophic methanogenesis [17] |
| *Methanolobus* | Halobacteriota | Anaerobic hypersaline environments (i.e. salt lakes, wastewater digesters) [18] | Methylotrophic methanogenesis [18] |
| *Methanomassiliicoccus* | Thermoplasmatota | Anaerobic environments (i.e. wetlands, human intestinal tract) [19] | Methylotrophic methanogenesis [20] |
| *Methanothermobacter* | Methanobacteriota | Thermophilic anaerobic environments (i.e. hot springs, geothermal soils) [21] | Hydrogenotrophic methanogenesis [22] |
| *Methanothrix* | Halobacteriota | Anaerobic environments (i.e. freshwaters, wetlands, rice paddies, oil resservoirs,permafrost soils, marine sediments) [23] | Acetoclastic methanogenesis [23] |
| **Bacteria** | | | |
| *Acetomicrobium* | Synergistota | Anaerobic thermophilic environments (i.e. oil reservoirs and associated production waters) [24] | Acetogenesis [25] |
| *Aliifodinibius* | Balneolota | Halophilic aerobic environment (i.e. salt mine) [26] | Chemoorganotrophy [27] |
| *Arhodomonas* | Pseudomonadota | Hypersaline environments (i.e. salt lakes, pertoleum reservoirs, subterranean brines) [28] | Chemoorganotrophy [28] |
| *Burkholderia-Caballeronia-Paraburkholderia* | Pseudomonadota | Ubiquitous [29] | Degradation of organic compounds [29] |
| *Coprothermobacter* | Coprothermobacterota | Thermophilic anaerobic environments [30] | Fermentation [30] |
| *Curvibacter* | Pseudomonadota | Freshwater environments (i.e. groundwater, wells, aquifers, oligotrophic lakes) [31,32] | Chemoorganotrophy [31,32] |
| *Dyella* | Pseudomonadota | Terrestrial soil environments [33] | Degradation of organic compounds [33] |
| *Glycocaulis* | Pseudomonadota | Aerobic marine environments (i.e. coastal seawater, deep-sea ecosystems) [34] | Chemoorganotrophy [34] |
| *Halomonas* | Pseudomonadota | Hypersaline environments (i.e. marine waters, salt lakes, saline soils) [35] | Chemoorganotrophy [36] |
| *Incertae Sedis* | Candidatus Eremiobacterota | Extreme environments [37] | Unknown* |
| *Marinilabilia* | Bacteroidota | Saline nevironments (i.e. marien sediments, coastal zones) [38] | Degradation of organic compounds [38] |
| *ML310M-34* | Balneolota | Unknown* | Unknown* |
| *Noviherbaspirillum* | Pseudomonadota | Aerobic environments [39] | Aerobic heterotrophy [39] |
| *Petrotoga* | Thermotogota | Thermophilic halophilic environments [40] | Fermentation [41] |
| *Pseudothermotoga* | Thermotogota | Thermophilic anaerobic environments [42] | Fermentation [43] |
| *Ralstonia* | Pseudomonadota | Various environments (i.e. soil, water, plant, humans) [44] | Various [45] |
| *Rhodohalobacter* | Balneolota | Saline environments [46] | Chemoorganotrophy [47] |
| *Salibacter* | Bacteroidota | Anaerobic halophilic environments [48] | Degradation of organic compounds [49] |
| *Sporomusa* | Bacillota | Anaerobic environments [50] | Fermentation [50] |
| *Sporosalibacterium* | Bacillota | Anaerobic halophilic environments (i.e. oil-contaminated soils, petroleum reservoirs, gold mines) [51] | Chemoorganotrophy [52] |
| *Syntrophus* | Thermodesulfobacteriota | Anaerobic environments (i.e. wastewaters, methanogenic digesters) [53] | Syntrophic bacteria [53] |
| *Tangfeifania* | Bacteroidota | Halophilic environments (i.e. lake sediments) [54] | Unknown* |
| *Thermovirga* | Synergistota | Thermophilic anaerobic environments (i.e. oil reservoirs) [55] | Fermentation [55] |
| *Unidentified* | Balneolota | Unknown* | Unknown* |
| *Urania-1B-19marine sedimen tgroup* | Planctomycetota | Unknown* | Unknown* |
| *Variovorax* | Pseudomonadota | Aerobic environments (i.e. soil, freswater) [56] | Chemoorganotrophy [56] |
| *Wenzhouxiangella* | Pseudomonadota | Marine environments (i.e. coastal sediment) [57] | Chemoorganotrophy [58] |

**Fig. S11**	Redundancy analysis (RDA) showing relationships between microbial community composition and physicochemical parameters across reservoir samples. Sites are represented by points and colored according to Clusters determined by NMDS (Cluster A in red, Cluster B in yellow and Cluster C in green). Arrows represent environmental variables, with arrow length indicating the strength of correlation with ordination axes. Genera are indicated by black points. The first two canonical axes explain 17.8% and 11.5% of the environmentally constrained variance, respectively.

**Table S8**	Results of Spearman correlation tests (*p-value* and rho value) performed between archaeal ecological characteristics and physico-chemical parameters. Values in bold correspond to significant values (*p-value* < 0.05). When *NA* is indicated, this means that there was insufficient data to perform the Spearman test.

| **Metabolism** | **Parameter** | **Ca** | **Cl** | **DOC** | **Fe** | **K** | **Mg** | **Mn** | **Na** | **NH_4_** | **NO_2_** | **NO_3_** | **pH** | **PO_4_** | **S_2_** | **Salinity** | **SO_4_** | **Temperature** | **TIC** |
| --- | --- | --- | --- | --- | --- | --- | --- | --- | --- | --- | --- | --- | --- | --- | --- | --- | --- | --- | --- |
| **Acetoclastic methanogenesis** | Rho | -0.37 | -0.43 | 0.46 | 0.37 | 0.33 | -0.23 | -0.50 | -0.47 | 0.39 | 0.43 | -0.22 | 0.19 | 0.50 | 0.62 | -0.44 | -0.32 | 0.21 | 0.68 |
|  | p-value | 0.14 | 0.07 | 0.10 | 0.11 | 0.16 | 0.37 | 0.12 | **0.04** | 0.19 | 0.40 | 0.68 | 0.44 | 0.25 | **0.02** | 0.06 | 0.18 | 0.39 | **0.01** |
| **Chemoorganotrophy** | Rho | 0.27 | 0.33 | -0.62 | -0.34 | 0.25 | 0.38 | -0.12 | 0.45 | -0.09 | NA | NA | -0.38 | NA | -0.24 | 0.38 | 0.30 | -0.47 | -0.12 |
|  | p-value | 0.12 | **0.04** | **<0.01** | **0.04** | 0.13 | **0.03** | **<0.01** | **<0.01** | 0.67 | NA | NA | **0.02** | NA | 0.22 | **0.02** | 0.06 | **<0.01** | 0.52 |
| **Fermentation** | Rho | 0.18 | 0.18 | -0.61 | -0.53 | 0.15 | 0.33 | -0.36 | 0.41 | -0.17 | NA | NA | -0.39 | NA | -0.21 | 0.25 | 0.19 | -0.41 | -0.22 |
|  | p-value | 0.48 | 0.45 | **0.02** | **0.02** | 0.53 | 0.19 | 0.27 | 0.08 | 0.57 | NA | NA | 0.10 | NA | 0.47 | 0.31 | 0.43 | 0.08 | 0.42 |
| **Hydrogenotrophic methanogenesis** | Rho | -0.17 | -0.39 | 0.39 | 0.35 | 0.18 | -0.02 | -0.28 | -0.40 | 0.17 | -0.05 | -0.46 | 0.09 | 0.24 | 0.46 | -0.43 | -0.34 | 0.16 | 0.41 |
|  | p-value | 0.24 | **<0.01** | **0.01** | **0.01** | 0.18 | 0.88 | 0.11 | **<0.01** | 0.31 | 0.86 | 0.06 | 0.50 | 0.29 | **<0.01** | **<0.01** | **0.01** | 0.24 | **0.01** |
| **Methylotrophic methanogenesis** | Rho | 0.00 | -0.13 | 0.11 | 0.11 | -0.15 | 0.13 | 0.14 | -0.12 | -0.04 | 0.09 | 0.12 | -0.08 | 0.02 | 0.06 | -0.12 | -0.06 | 0.20 | 0.09 |
|  | p-value | 0.99 | 0.25 | 0.41 | 0.35 | 0.20 | 0.30 | 0.37 | 0.32 | 0.78 | 0.68 | 0.59 | 0.48 | 0.94 | 0.66 | 0.28 | 0.63 | 0.09 | 0.50 |
| **Sulfur respiration** | Rho | 0.20 | 0.39 | -0.24 | 0.00 | 0.26 | 0.15 | -0.10 | 0.39 | -0.23 | NA | NA | -0.17 | NA | -0.14 | 0.39 | 0.39 | -0.22 | -0.06 |
|  | p-value | 0.43 | 0.10 | 0.41 | 1.00 | 0.29 | 0.56 | 0.77 | 0.10 | 0.45 | NA | NA | 0.48 | NA | 0.62 | 0.10 | 0.10 | 0.38 | 0.83 |
| **Symbiotic archaea** | Rho | -0.33 | 0.28 | NA | -0.11 | -0.29 | -0.47 | 0.27 | 0.21 | 0.25 | NA | NA | 0.67 | -0.64 | NA | 0.23 | 0.40 | -0.40 | NA |
|  | p-value | 0.19 | 0.24 | NA | 0.65 | 0.22 | 0.06 | 0.43 | 0.40 | 0.41 | NA | NA | **<0.01** | 0.13 | NA | 0.34 | 0.09 | 0.09 | NA |
| **Unknown** | Rho | 0.26 | 0.31 | -0.63 | -0.39 | 0.24 | 0.39 | -0.17 | 0.44 | -0.08 | NA | NA | -0.37 | NA | -0.24 | 0.36 | 0.27 | -0.47 | -0.14 |
|  | p-value | **0.03** | **0.01** | **<0.01** | **<0.01** | **0.04** | **<0.01** | 0.26 | **<0.01** | 0.56 | NA | NA | 0.00 | NA | 0.07 | **<0.01** | **0.02** | **<0.01** | 0.27 |

**Table S9**	Results of Spearman correlation tests (*p-value* and rho value) performed between bacterial ecological characteristic and physico-chemical parameters. Values in bold correspond to significant values (*p-value* < 0.05). When *NA* is indicated, this means that there was insufficient data to perform the Spearman test.

| **Metabolism** | **Parameter** | **Ca** | **Cl** | **DOC** | **Fe** | **K** | **Mg** | **Mn** | **Na** | **NH_4_** | **NO_2_** | **NO_3_** | **pH** | **PO_4_** | **S_2_** | **Salinity** | **SO_4_** | **Temperature** | **TIC** |
| --- | --- | --- | --- | --- | --- | --- | --- | --- | --- | --- | --- | --- | --- | --- | --- | --- | --- | --- | --- |
| **Acetogenesis** | Rho | -0.14 | -0.53 | 0.65 | 0.53 | 0.24 | 0.09 | -0.27 | -0.55 | 0.41 | 0.55 | -0.46 | 0.03 | 0.37 | 0.57 | -0.55 | -0.42 | 0.40 | 0.53 |
|  | p-value | 0.58 | **0.02** | **0.01** | **0.02** | 0.32 | 0.73 | 0.42 | **0.01** | 0.16 | 0.26 | 0.35 | 0.91 | 0.41 | **0.03** | **0.02** | 0.07 | 0.09 | **0.04** |
| **Aerobic heterotrophy** | Rho | 0.41 | 0.20 | -0.03 | 0.27 | 0.13 | 0.48 | 0.40 | 0.15 | 0.31 | -0.53 | -0.53 | -0.27 | -0.21 | -0.21 | 0.20 | -0.08 | -0.07 | -0.11 |
|  | p-value | 0.10 | 0.41 | 0.91 | 0.26 | 0.58 | 0.05 | 0.22 | 0.55 | 0.30 | 0.28 | 0.28 | 0.26 | 0.65 | 0.47 | 0.41 | 0.75 | 0.77 | 0.68 |
| **Chemoorganotrophy** | Rho | -0.10 | 0.21 | -0.22 | -0.19 | -0.14 | -0.20 | 0.13 | 0.16 | 0.17 | 0.17 | 0.30 | 0.22 | -0.40 | -0.14 | 0.19 | 0.24 | -0.15 | -0.21 |
|  | p-value | 0.20 | **0.01** | **0.01** | **0.01** | 0.07 | **0.01** | 0.22 | 0.04 | 0.07 | 0.21 | **0.03** | **<0.01** | **<0.01** | 0.11 | **0.01** | **<0.01** | **0.05** | **0.02** |
| **Degradation of organic compounds** | Rho | 0.07 | -0.04 | 0.02 | 0.06 | -0.07 | -0.12 | 0.15 | -0.07 | -0.06 | -0.25 | 0.25 | -0.02 | -0.28 | -0.18 | -0.05 | -0.19 | 0.13 | -0.45 |
|  | p-value | 0.58 | 0.76 | 0.89 | 0.61 | 0.55 | 0.33 | 0.32 | 0.53 | 0.65 | 0.24 | 0.23 | 0.86 | 0.15 | 0.19 | 0.68 | 0.09 | 0.27 | **<0.01** |
| **Fermentation** | Rho | -0.15 | -0.40 | 0.46 | 0.39 | 0.23 | -0.04 | -0.31 | -0.43 | 0.24 | 0.19 | -0.28 | 0.03 | 0.34 | 0.42 | -0.40 | -0.37 | 0.34 | 0.33 |
|  | p-value | 0.16 | **<0.01** | **<0.01** | **<0.01** | **0.02** | 0.70 | **0.02** | **<0.01** | 0.05 | 0.32 | 0.14 | 0.77 | **0.04** | **<0.01** | **<0.01** | **<0.01** | **<0.01** | **<0.01** |
| **Syntrophic bacteria** | Rho | -0.10 | 0.00 | -0.25 | 0.03 | -0.09 | -0.01 | 0.09 | 0.00 | -0.03 | 0.13 | 0.40 | 0.05 | 0.21 | 0.24 | 0.00 | 0.23 | -0.15 | 0.15 |
|  | p-value | 0.71 | 0.99 | 0.40 | 0.89 | 0.73 | 0.96 | 0.78 | 0.99 | 0.91 | 0.80 | 0.43 | 0.85 | 0.65 | 0.41 | 0.99 | 0.33 | 0.54 | 0.59 |
| **Unknown** | Rho | -0.09 | 0.27 | 0.13 | -0.10 | -0.25 | -0.23 | 0.28 | 0.20 | 0.34 | -0.25 | 0.03 | 0.33 | -0.52 | -0.12 | 0.23 | 0.29 | -0.07 | -0.26 |
|  | p-value | 0.46 | **0.02** | 0.34 | 0.38 | **0.03** | 0.06 | 0.07 | 0.09 | **0.01** | 0.24 | 0.90 | **<0.01** | **<0.01** | 0.37 | **0.04** | **0.01** | 0.53 | **0.04** |
| **Various** | Rho | 0.07 | -0.23 | -0.14 | -0.05 | 0.00 | -0.28 | 0.13 | -0.30 | -0.24 | NA | 0.66 | -0.23 | NA | -0.21 | -0.23 | -0.50 | 0.33 | -0.59 |
|  | p-value | 0.79 | 0.34 | 0.62 | 0.85 | 0.99 | 0.27 | 0.69 | 0.21 | 0.42 | NA | 0.15 | 0.34 | NA | 0.47 | 0.34 | 0.03 | 0.16 | **0.02** |

**References**

[1]	Oren A. Candidatus Haloredivivus. Bergey’s Manual of Systematics of Archaea and Bacteria, 2020, p. 1–4. https://doi.org/10.1002/9781118960608.gbm01992.

[2]	Lang K, Schuldes J, Klingl A, Poehlein A, Daniel R, Brune A. New Mode of Energy Metabolism in the Seventh Order of Methanogens as Revealed by Comparative Genome Analysis of “Candidatus Methanoplasma termitum.” Applied and Environmental Microbiology 2015;81:1338–52. https://doi.org/10.1128/AEM.03389-14.

[3]	Sorokin DY, Kublanov IV, Yakimov MM, Rijpstra WIC, Sinninghe Damsté JS. Halanaeroarchaeum sulfurireducens gen. nov., sp. nov., the first obligately anaerobic sulfur-respiring haloarchaeon, isolated from a hypersaline lake. Int J Syst Evol Microbiol 2016;66:2377–81. https://doi.org/10.1099/ijsem.0.001041.

[4]	Minegishi H, Echigo A, Nagaoka S, Kamekura M, Usami R. Halarchaeum acidiphilum gen. nov., sp. nov., a moderately acidophilic haloarchaeon isolated from commercial solar salt. International Journal of Systematic and Evolutionary Microbiology 2010;60:2513–6. https://doi.org/10.1099/ijs.0.013722-0.

[5]	Cui H-L. Halobellus. Bergey’s Manual of Systematics of Archaea and Bacteria, John Wiley & Sons, Ltd; 2016, p. 1–13. https://doi.org/10.1002/9781118960608.gbm01335.

[6]	Cui H-L, Gao X, Yang X, Xu X-W. Halolamina pelagica gen. nov., sp. nov., a new member of the family Halobacteriaceae. International Journal of Systematic and Evolutionary Microbiology 2011;61:1617–21. https://doi.org/10.1099/ijs.0.026799-0.

[7]	Kondo Y, Minegishi H, Echigo A, Shimane Y, Kamekura M, Itoh T, Ohkuma M, Tanaka A, Takahashi-Ando N, Fukushima Y, Yoshida Y, Ihara K, Usami R. Haloparvum alkalitolerans sp. nov., alkali-tolerant haloarchaeon isolated from commercial salt. International Journal of Systematic and Evolutionary Microbiology 2016;66:5314–9. https://doi.org/10.1099/ijsem.0.001513.

[8]	Amoozegar MA, Makhdoumi-Kakhki A, Shahzadeh Fazeli SA, Azarbaijani R, Ventosa A. Halopenitus persicus gen. nov., sp. nov., an archaeon from an inland salt lake. Int J Syst Evol Microbiol 2012;62:1932–6. https://doi.org/10.1099/ijs.0.036921-0.

[9]	La Cono V, Messina E, Rohde M, Arcadi E, Ciordia S, Crisafi F, Denaro R, Ferrer M, Giuliano L, Golyshin PN, Golyshina OV, Hallsworth JE, La Spada G, Mena MC, Merkel AY, Shevchenko MA, Smedile F, Sorokin DY, Toshchakov SV, Yakimov MM. Symbiosis between nanohaloarchaeon and haloarchaeon is based on utilization of different polysaccharides. Proceedings of the National Academy of Sciences 2020;117:20223–34. https://doi.org/10.1073/pnas.2007232117.

[10]	La Cono V, Messina E, Rohde M, Arcadi E, Ciordia S, Crisafi F, Denaro R, Ferrer M, Giuliano L, Golyshin PN, Golyshina OV, Hallsworth JE, La Spada G, Mena MC, Merkel AY, Shevchenko MA, Smedile F, Sorokin DY, Toshchakov SV, Yakimov MM. Symbiosis between nanohaloarchaeon and haloarchaeon is based on utilization of different polysaccharides. Proceedings of the National Academy of Sciences 2020;117:20223–34. https://doi.org/10.1073/pnas.2007232117.

[11]	Munson-McGee JH, Field EK, Bateson M, Rooney C, Stepanauskas R, Young MJ. Nanoarchaeota, Their Sulfolobales Host, and Nanoarchaeota Virus Distribution across Yellowstone National Park Hot Springs. Applied and Environmental Microbiology 2015;81:7860–8. https://doi.org/10.1128/AEM.01539-15.

[12]	Jarett J, Nayfach S, Podar M, Inskeep W, Ivanova N, Munson-McGee J, Schulz F, Young M, Jay Z, Beam J, Kyrpides N, Malmstrom R, Stepanauskas R, Woyke T. Single-cell genomics of co-sorted Nanoarchaeota suggests novel putative host associations and diversification of proteins involved in symbiosis. Microbiome 2018;6. https://doi.org/10.1186/s40168-018-0539-8.

[13]	Chellapandi P, Bharathi M, Sangavai C, Prathiviraj R. *Methanobacterium formicicum* as a target rumen methanogen for the development of new methane mitigation interventions: A review. Veterinary and Animal Science 2018;6:86–94. https://doi.org/10.1016/j.vas.2018.09.001.

[14]	Dy S, B A, Ay M, Wic R, Jss D, Mv S, Mcm van L. Methanosalsum natronophilum sp. nov., and Methanocalculus alkaliphilus sp. nov., haloalkaliphilic methanogens from hypersaline soda lakes. International Journal of Systematic and Evolutionary Microbiology 2015;65. https://doi.org/10.1099/ijsem.0.000488.

[15]	Ollivier B, Fardeau M-L, Cayol J-L, Magot M, Patel BKC, Prensier G, Garcia J-L. Methanocalculus halotolerans gen. nov., sp. nov., isolated from an oil-producing well. International Journal of Systematic and Evolutionary Microbiology 1998;48:821–8. https://doi.org/10.1099/00207713-48-3-821.

[16]	Zhilina T. Methanohalobium. Bergey’s Manual of Systematics of Archaea and Bacteria, 2015. https://doi.org/10.1002/9781118960608.gbm00515.

[17]	Bueno de Mesquita CP, Wu D, Tringe SG. Methyl-Based Methanogenesis: an Ecological and Genomic Review. Microbiology and Molecular Biology Reviews 2023;87:e00024-22. https://doi.org/10.1128/mmbr.00024-22.

[18]	Tsola SL, Zhu Y, Chen Y, Sanders IA, Economou CK, Brüchert V, Eyice Ö. Methanolobus use unspecific methyltransferases to produce methane from dimethylsulphide in Baltic Sea sediments. Microbiome 2024;12:3. https://doi.org/10.1186/s40168-023-01720-w.

[19]	Weil M, Hoff KJ, Meißner W, Schäfer F, Söllinger A, Wang H, Hagenau L, Kuss AW, Urich T. Full Genome Sequence of a Methanomassiliicoccales Representative Enriched from Peat Soil. Microbiology Resource Announcements 2021;10:e00443-21. https://doi.org/10.1128/MRA.00443-21.

[20]	Borrel G, Harris HMB, Parisot N, Gaci N, Tottey W, Mihajlovski A, Deane J, Gribaldo S, Bardot O, Peyretaillade E, Peyret P, O’Toole PW, Brugère J-F. Genome Sequence of “Candidatus Methanomassiliicoccus intestinalis” Issoire-Mx1, a Third Thermoplasmatales-Related Methanogenic Archaeon from Human Feces. Genome Announc 2013;1:e00453-13. https://doi.org/10.1128/genomeA.00453-13.

[21]	McKay LJ, Klingelsmith KB, Deutschbauer AM, Inskeep WP, Fields MW. Draft Genome Sequence of Methanothermobacter thermautotrophicus WHS, a Thermophilic Hydrogenotrophic Methanogen from Washburn Hot Springs in Yellowstone National Park, USA. Microbiology Resource Announcements 2021;10:e01157. https://doi.org/10.1128/MRA.01157-20.

[22]	Kaster A-K, Goenrich M, Seedorf H, Liesegang H, Wollherr A, Gottschalk G, Thauer RK. More Than 200 Genes Required for Methane Formation from H2 and CO2 and Energy Conservation Are Present in Methanothermobacter marburgensis and Methanothermobacter thermautotrophicus. Archaea 2011;2011:973848. https://doi.org/10.1155/2011/973848.

[23]	Zhou J, Smith JA, Li M, Holmes DE. Methane production by Methanothrix thermoacetophila via direct interspecies electron transfer with Geobacter metallireducens. mBio 2023;14:e00360-23. https://doi.org/10.1128/mbio.00360-23.

[24]	Hania WB, Bouanane-Darenfed A, Cayol J-L, Ollivier B, Fardeau M-L. Reclassification of Anaerobaculum mobile, Anaerobaculum thermoterrenum, Anaerobaculum hydrogeniformans as Acetomicrobium mobile comb. nov., Acetomicrobium thermoterrenum comb. nov. and Acetomicrobium hydrogeniformans comb. nov., respectively, and emendation of the genus Acetomicrobium. International Journal of Systematic and Evolutionary Microbiology 2016;66:1506–9. https://doi.org/10.1099/ijsem.0.000910.

[25]	Schut GJ, Thorgersen MP, Poole FL, Haja DK, Putumbaka S, Adams MWW. Tungsten enzymes play a role in detoxifying food and antimicrobial aldehydes in the human gut microbiome. Proceedings of the National Academy of Sciences 2021;118:e2109008118. https://doi.org/10.1073/pnas.2109008118.

[26]	Wang Y-X, Liu J-H, Xiao W, Ma X-L, Lai Y-H, Li Z-Y, Ji K-Y, Wen M-L, Cui X-L. Aliifodinibius roseus gen. nov., sp. nov., and Aliifodinibius sediminis sp. nov., two moderately halophilic bacteria isolated from salt mine samples. International Journal of Systematic and Evolutionary Microbiology 2013;63:2907–13. https://doi.org/10.1099/ijs.0.043869-0.

[27]	Hahnke RL, Meier-Kolthoff JP, García-López M, Mukherjee S, Huntemann M, Ivanova NN, Woyke T, Kyrpides NC, Klenk H-P, Göker M. Genome-Based Taxonomic Classification of Bacteroidetes. Front Microbiol 2016;7:2003. https://doi.org/10.3389/fmicb.2016.02003.

[28]	Adkins JP, Madigan MT, Mandelco L, Woese CR, Tanner RS. Arhodomonas aquaeolei gen. nov., sp. nov., an Aerobic, Halophilic Bacterium Isolated from a Subterranean Brine. International Journal of Systematic and Evolutionary Microbiology 1993;43:514–20. https://doi.org/10.1099/00207713-43-3-514.

[29]	Bellés-Sancho P, Beukes C, James EK, Pessi G. Nitrogen-Fixing Symbiotic Paraburkholderia Species: Current Knowledge and Future Perspectives. Nitrogen 2023;4:135–58. https://doi.org/10.3390/nitrogen4010010.

[30]	Pavan ME, Pavan EE, Glaeser SP, Etchebehere C, Kämpfer P, Pettinari MJ, López NI. Proposal for a new classification of a deep branching bacterial phylogenetic lineage: transfer of Coprothermobacter proteolyticus and Coprothermobacter platensis to Coprothermobacteraceae fam. nov., within Coprothermobacterales ord. nov., Coprothermobacteria classis nov. and Coprothermobacterota phyl. nov. and emended description of the family Thermodesulfobiaceae. International Journal of Systematic and Evolutionary Microbiology 2018;68:1627–32. https://doi.org/10.1099/ijsem.0.002720.

[31]	Ding L, Yokota A. Curvibacter fontana sp. nov., a microaerobic bacteria isolated from well water. J Gen Appl Microbiol 2010;56:267–71. https://doi.org/10.2323/jgam.56.267.

[32]	Ding L, Yokota A. Proposals of Curvibacter gracilis gen. nov., sp. nov. and Herbaspirillum putei sp. nov. for bacterial strains isolated from well water and reclassification of [Pseudomonas] huttiensis, [Pseudomonas] lanceolata, [Aquaspirillum] delicatum and [Aquaspirillum] autotrophicum as Herbaspirillum huttiense comb. nov., Curvibacter lanceolatus comb. nov., Curvibacter delicatus comb. nov. and Herbaspirillum autotrophicum comb. nov. International Journal of Systematic and Evolutionary Microbiology 2004;54:2223–30. https://doi.org/10.1099/ijs.0.02975-0.

[33]	Xie C-H, Yokota A. Dyella japonica gen. nov., sp. nov., a γ-proteobacterium isolated from soil. International Journal of Systematic and Evolutionary Microbiology 2005;55:753–6. https://doi.org/10.1099/ijs.0.63377-0.

[34]	Abraham W-R, Lünsdorf H, Vancanneyt M, Smit J. Cauliform bacteria lacking phospholipids from an abyssal hydrothermal vent: proposal of Glycocaulis abyssi gen. nov., sp. nov., belonging to the family Hyphomonadaceae. International Journal of Systematic and Evolutionary Microbiology 2013;63:2207–15. https://doi.org/10.1099/ijs.0.047894-0.

[35]	Gaboyer F, Vandenabeele-Trambouze O, Cao J, Ciobanu M-C, Jebbar M, Le Romancer M, Alain K. Physiological features of *Halomonas lionensis* sp. nov., a novel bacterium isolated from a Mediterranean Sea sediment. Research in Microbiology 2014;165:490–500. https://doi.org/10.1016/j.resmic.2014.07.009.

[36]	Ventosa A, de la Haba RR, Arahal DR, Sánchez-Porro C. Halomonas. Bergey’s Manual of Systematics of Archaea and Bacteria, John Wiley & Sons, Ltd; 2021, p. 1–111. https://doi.org/10.1002/9781118960608.gbm01190.pub2.

[37]	Ji M, Williams TJ, Montgomery K, Wong HL, Zaugg J, Berengut JF, Bissett A, Chuvochina M, Hugenholtz P, Ferrari BC. Candidatus Eremiobacterota, a metabolically and phylogenetically diverse terrestrial phylum with acid-tolerant adaptations. ISME J 2021;15:2692–707. https://doi.org/10.1038/s41396-021-00944-8.

[38]	Zhang H-Z, Teng J-H, Zhou H-Y, Liu T-H, Lu D-C, Du Z-J. Six new bacterial species of Marinilabiliales isolated from the marine coastal sediment and reclassified Ancylomarina and Labilibaculum as Marinifilum comb. nov. based on the genome analysis. Front Microbiol 2025;16:1634775. https://doi.org/10.3389/fmicb.2025.1634775.

[39]	Lin S-Y, Hameed A, Arun AB, Liu Y-C, Hsu Y-H, Lai W-A, Rekha PD, Young C-C. Description of Noviherbaspirillum malthae gen. nov., sp. nov., isolated from an oil-contaminated soil, and proposal to reclassify Herbaspirillum soli, Herbaspirillum aurantiacum, Herbaspirillum canariense and Herbaspirillum psychrotolerans as Noviherbaspirillum soli comb. nov., Noviherbaspirillum aurantiacum comb. nov., Noviherbaspirillum canariense comb. nov. and Noviherbaspirillum psychrotolerans comb. nov. based on polyphasic analysis. Int J Syst Evol Microbiol 2013;63:4100–7. https://doi.org/10.1099/ijs.0.048231-0.

[40]	Miranda-Tello E, Fardeau M-L, Joulian C, Magot M, Thomas P, Tholozan J-L, Ollivier B. Petrotoga halophila sp. nov., a thermophilic, moderately halophilic, fermentative bacterium isolated from an offshore oil well in Congo. Int J Syst Evol Microbiol 2007;57:40–4. https://doi.org/10.1099/ijs.0.64516-0.

[41]	Miranda-Tello E, Fardeau M-L, Joulian C, Magot M, Thomas P, Tholozan JL, Ollivier B. Petrotoga halophila sp nov., a thermophilic, moderately halophilic, fermentative bacterium isolated from an offshore oil well in Congo. International Journal of Systematic and Evolutionary Microbiology 2007;57. https://doi.org/10.1099/ijs.0.64516-0.

[42]	Roumagnac M, Pradel N, Bartoli M, Garel M, Jones AA, Armougom F, Fenouil R, Tamburini C, Ollivier B, Summers ZM, Dolla A. Responses to the Hydrostatic Pressure of Surface and Subsurface Strains of Pseudothermotoga elfii Revealing the Piezophilic Nature of the Strain Originating From an Oil-Producing Well. Front Microbiol 2020;11:588771. https://doi.org/10.3389/fmicb.2020.588771.

[43]	Ravot G, Magot M, Fardeau M-L, Patel BKC, Prensier G, Egan A, Garcia J-L, Ollivier B. Thermotoga elfii sp. nov., a Novel Thermophilic Bacterium from an African Oil-Producing Well. International Journal of Systematic and Evolutionary Microbiology 1995;45:308–14. https://doi.org/10.1099/00207713-45-2-308.

[44]	Lu C-H, Zhang Y-Y, Jiang N, Chen W, Shao X, Zhao Z-M, Lu W-L, Hu X, Xi Y-X, Zou S-Y, Wei Q-J, Lin Z-L, Gong L, Gai X-T, Zhang L-Q, Li J-Y, Jin Y, Xia Z-Y. Ralstonia chuxiongensis sp. nov., Ralstonia mojiangensis sp. nov., and Ralstonia soli sp. nov., isolated from tobacco fields, are three novel species in the family Burkholderiaceae. Front Microbiol 2023;14. https://doi.org/10.3389/fmicb.2023.1179087.

[45]	Rivera-Zuluaga K, Hiles R, Barua P, Caldwell D, Iyer-Pascuzzi AS. Getting to the root of *Ralstonia* invasion. Seminars in Cell & Developmental Biology 2023;148–149:3–12. https://doi.org/10.1016/j.semcdb.2022.12.002.

[46]	Xia J, Xie Z-H, Dunlap CA, Rooney AP, Du Z-J. Rhodohalobacter halophilus gen. nov., sp. nov., a moderately halophilic member of the family Balneolaceae. International Journal of Systematic and Evolutionary Microbiology 2017;67:1281–7. https://doi.org/10.1099/ijsem.0.001806.

[47]	Han S-B, Yu Y-H, Ju Z, Li Y, Zhang R, Hou X-J, Ma X-Y, Yu X-Y, Sun C, Wu M. Rhodohalobacter barkolensis sp. nov., isolated from a saline lake and emended description of the genus Rhodohalobacter. Int J Syst Evol Microbiol 2018;68:1949–54. https://doi.org/10.1099/ijsem.0.002775.

[48]	Lu D-C, Xia J, Dunlap CA, Rooney AP, Du Z-J. Salibacter halophilus gen. nov., sp. nov., isolated from a saltern. Int J Syst Evol Microbiol 2017;67:1784–8. https://doi.org/10.1099/ijsem.0.001807.

[49]	Lu D, Xia J, Dunlap C, Rooney A, Du Z. Salibacter halophilus gen. nov., sp. nov., isolated from a saltern. International Journal of Systematic and Evolutionary Microbiology 2017;67. https://doi.org/10.1099/ijsem.0.001807.

[50]	Möller B, Oßmer R, Howard BH, Gottschalk G, Hippe H. Sporomusa, a new genus of gram-negative anaerobic bacteria including Sporomusa sphaeroides spec. nov. and Sporomusa ovata spec. nov. Arch Microbiol 1984;139:388–96. https://doi.org/10.1007/BF00408385.

[51]	Rezgui R, Ben Ali Gam Z, Ben Hamed S, Fardeau M-L, Cayol J-L, Maaroufi A, Labat M. Sporosalibacterium faouarense gen. nov., sp. nov., a moderately halophilic bacterium isolated from oil-contaminated soil. Int J Syst Evol Microbiol 2011;61:99–104. https://doi.org/10.1099/ijs.0.017715-0.

[52]	Rezgui R, Ben Ali Gam Z, Ben Hamed S, Fardeau M-L, Cayol J-L, Maaroufi A, Labat M. Sporosalibacterium faouarense gen. nov., sp. nov., a moderately halophilic bacterium isolated from oil-contaminated soil. International Journal of Systematic and Evolutionary Microbiology 2011;61:99–104. https://doi.org/10.1099/ijs.0.017715-0.

[53]	McInerney MJ, Rohlin L, Mouttaki H, Kim U, Krupp RS, Rios-Hernandez L, Sieber J, Struchtemeyer CG, Bhattacharyya A, Campbell JW, Gunsalus RP. The genome of Syntrophus aciditrophicus: Life at the thermodynamic limit of microbial growth. Proceedings of the National Academy of Sciences 2007;104:7600–5. https://doi.org/10.1073/pnas.0610456104.

[54]	Liu Q-Q, Li X-L, Rooney AP, Du Z-J, Chen G-J. Tangfeifania diversioriginum gen. nov., sp. nov., a representative of the family Draconibacteriaceae. International Journal of Systematic and Evolutionary Microbiology 2014;64:3473–7. https://doi.org/10.1099/ijs.0.066902-0.

[55]	Dahle H, Birkeland N-K. Thermovirga lienii gen. nov., sp. nov., a novel moderately thermophilic, anaerobic, amino-acid-degrading bacterium isolated from a North Sea oil well. Int J Syst Evol Microbiol 2006;56:1539–45. https://doi.org/10.1099/ijs.0.63894-0.

[56]	Decewicz P, Kitowicz M, Radlinska M. Characteristics and Comparative Genomic Analysis of a Novel Virus, VarioGold, the First Bacteriophage of Variovorax. Int J Mol Sci 2022;23:13539. https://doi.org/10.3390/ijms232113539.

[57]	Guo L-Y, Dunlap CA, Rooney AP, Chen G-J, Du Z-J. Wenzhouxiangella sediminis sp. nov., isolated from coastal sediment. International Journal of Systematic and Evolutionary Microbiology 2016;66:4575–9. https://doi.org/10.1099/ijsem.0.001393.

[58]	Wang G, Tang M, Li T, Dai S, Wu H, Chen C, He H, Fan J, Xiang W, Li X. Wenzhouxiangella marina gen. nov, sp. nov, a marine bacterium from the culture broth of Picochlorum sp. 122, and proposal of Wenzhouxiangellaceae fam. nov. in the order Chromatiales. Antonie van Leeuwenhoek 2015;107:1625–32. https://doi.org/10.1007/s10482-015-0458-7.
